# Accommodation of Biodiversity in Swedish Municipalities. A National Survey

**DOI:** 10.1007/s00267-026-02429-w

**Published:** 2026-04-10

**Authors:** Lisbet Christoffersen, Jan-Eric Englund, Thomas Randrup

**Affiliations:** Swedish University of Agricultural Sciences, Alnarp, Sweden

**Keywords:** Views of nature, discourses, organisation, management trends

## Abstract

Habitat networks for biodiversity are increasingly degraded and fragmented. This study investigates how Swedish municipal landscape managers accommodate biodiversity in urban and peri-urban landscapes, with particular attention to the promotion of ‘wilder landscapes’. We explore their view of nature as related to wilder landscapes too. Drawing on a national survey with responses from 70% of all Swedish municipalities, we analyse how biodiversity initiatives are prioritised, organised, and implemented within local governments. The survey reveals a strong interest in biodiversity accommodation, reflected in a wide range of strategies and concrete actions. However, organisational constraints, limited political support, and funding shortages often result in fragmented and ad hoc approaches. Competing agendas, as expressed in different views of nature as well as in bound tasks of the public managers, may shape future directions in municipal landscape management, especially those emphasising multifunctional green spaces. We discuss the implications of municipal organisational structures, managerial perspectives, individual views of nature and institutional frameworks for biodiversity accommodation and offer projections for future municipal biodiversity strategies in Sweden.

## Introduction

In Sweden, like in other places, habitat networks for biodiversity are generally degraded (Dawson et al. 2017). The result is the absence of large intact nature areas and the loss of the compositional, structural and functional elements of biodiversity found in naturally dynamic landscapes. Simultaneously, urban areas’ built form is presumed to have the single most significant effect on ecosystems on land in Sweden (Andersson & Colding 2014) causing profound alterations of natural habitats^1^ due to densification and habitat fragmentation (Randrup et al. 2021).

Nevertheless, urban and peri-urban ecosystems contribute to biodiversity and are considered important for sustainable urban development (Rega-Brodsky et al. 2022; Oke et al. 2021), and their preservation and promotion are encouraged by the European Commission (2024) and the UN (2022). Moreover, urban areas are where people live, and their concern about other species is paramount of the attitude towards nature conservation overall (Hartig & Kahn 2016). To meet international and national environmental targets related to biodiversity protection, Hanson and Olsson (2023) found that ‘dense and green cities’ is an important planning paradigm of many Swedish municipalities. Biodiversity has apparently become an important planning goal, yet extinctions continue (Singleton 2023). In this study, to understand this paradox in more detail, we explore central measures taken by landscape managers across Swedish municipalities to accommodate biodiversity, and factors which prevent biodiversity goals from being reached. ‘Accommodation’ should be understood as the yielding and sharing of living space; in the context of our study, the explicit strategies and concrete measures that support and integrate (spaces for) biodiversity into planning and management of green areas. This is parallel to how Briggs (2010) frame biodiversity accommodation like a key ecological process facilitating species co-existence and diversification. While Sweden is the subject of analysis in our study, preventing factors may reflect common barriers at an international scale as well.

When looking at landscape management trends across Europe, the reservation of areas to nature’s own dynamics, i.e. deliberate efforts to facilitate natural processes (or non-human autonomy/agency), here referred to as ‘wilder landscapes’, has become an acknowledged biological approach (e.g. Pereira & Navarro 2015) to halt the biodiversity decline.

Urban wilderness, whether planned for or spontaneous, has become the subject of new management methods debates and experiments across Europe (Kowarik 2018; Jorgensen and Tylecote 2007; Knudsen et al. 2019; Müller et al. 2018; Sarabi et al. 2023). More ‘wildness’ in urban areas has even been subject to national competition between municipalities in Denmark, a challenge given by the Minister of the Environment and broadcasted at national media (Hvem er Danmarks vildeste kommune? - Vild Med Vilje).

In Sweden, forest is the dominating landscape type, also in peri-urban areas. Therefore, we have a special focus on forest management in addition to investigating landscape- and local level biodiversity accommodating measures. Urban forest account for 4,3 million hectares or 15% of all forest. Of this, 4 million hectares or 93%, is production forest, and far the most is privately owned. Municipalities and Counties own around 33% of the very “inner circle” forest, i.e. 300 m from the urbanised area, decreasing until 3000 m which is the defined limit for when a forest is “urban” (Skogsstyrelsen 2024). Although Swedish municipalities develop and politically adapt the ‘municipal comprehensive plan’ (MCP), a stationary overall planning tool, planning regarding forested land is largely absent in these (Ambjörnsson et al. 2016). Forestry is a profession, and forest management is sector-oriented, i.e. not locally based (Stjernström et al. 2018). Moreover, a gap in the MCP regarding access to knowledge about forest condition and forest land-use (ibid; Thellbro et al. 2017) affect the ability for the municipality to take planning action towards e.g. accommodation of biodiversity and climate adaptation on privately owned land. They can mainly plan on own municipal land. In municipal forests, a recreational focus since the 1970s and ecological considerations since the 1990s have produced enhanced levels of biodiversity in some areas (Rydberg & Falck 2000), a good baseline for pursuing ‘a wilder’ management approach.

Little is known about whether or to what extend the ‘wilder trend’ of deliberate efforts to facilitate biodiversity through natural processes has taken hold in municipal forest and landscape management in Sweden. Elander et al. (2005), studied biodiversity promotion and development in four Swedish cities. They found that then, biodiversity had a weak position in overall urban policy packages as well as among municipal planners and mangers. Biodiversity was most often connected to the recreational aspects of urban green structure and lacked strategic focus. In a more recent study, based on urban open space managers perceptions, Randrup et al. (2021) found that urban peripheries gradually have assumed a wilder expression, but this seems mostly to relate to limited management budgets, although biodiversity promotion can possibly be stated as a positive outcome. Therefore, we include questions about funding and organisation of biodiversity accommodating measures in the survey.

We assume that municipal landscape managers’ view of wilder landscapes to accommodate biodiversity affect both urban and peri-urban landscapes. We thus explore their views of nature, as conceived by Christoffersen and Randrup (2024) in their development of a typology including three ideal types of landscape managers, as part of our study.

### Objective with the study

The overall objective of our study is to understand landscape managers’ approaches as related to the promotion of wilder landscapes and the associated accommodation of biodiversity. We explore central measures being taken and factors which prevent biodiversity goals from being reached. We base our study on a national survey, and the following three research questions:

RQ1: What are the predominant types of biodiversity accommodating measures being taken in Swedish municipalities (past and present)?

RQ2: How is biodiversity funded and organised within municipalities?

RQ3: What are the fundamental views of nature among public landscape managers?

In the following, we first introduce our conceptual framework, then the methodology. The results of the survey make up the central part of the paper. The results are presented with only little analysis to clearly disclose the basis for our interpretation, which follows in a discussion of our key findings.

## Conceptual Framework

### Wilder Landscapes as Biodiversity Accommodating Approach?

Wilder landscapes, i.e. deliberate efforts to facilitate natural processes, have become prominent approaches to accommodate biodiversity (Pereira & Navarro 2015). In the British Isles, profoundly cultural land has been designated to nature’s own processes (Thomas 2022; Deary and Warren 2017), and in Denmark, a decision to establish 16 new ‘Nature National Parks’ including browsing animals such as horses, elk, bison and cattle, have been politically adopted (Buhl 2023), several close to urban areas, including the capital, Copenhagen.

The measures needed to create wilder landscapes have created disputes between proponents, land owners, managers and different landscape users (Pellis and Jong 2016; Lorimer 2015; Deary and Warren 2017), among other about how landscapes should be managed, by and for whom, about landscape ethics (Arler 2009) and aesthetics (Porteous 1996) as well as animal welfare (Lorimer 2015). A wilder landscape implies the acceptance of ‘non-utility’ and ‘non-conservation’ in the conventional sense, i.e. acceptance of a landscape expression and development resulting from natural dynamics (Christoffersen & Randrup 2024). This ‘loss of control’ causes concern. Those concerned with conservation fear the displacement of rare species and habitats when natural succession takes over (Drenthen, 2018), while specific urban concerns often involve the sense of safety (Maas et al. 2009; Wang et al. 2025), although wilder urban landscapes also arouse curiosity and excitement (Jorgensen and Tylecote 2007), indicating that preference and a sense of danger are not necessarily opposites, but relates to the diversity of preferences, represented by various user groups.

### The Views-of-Nature Typology

Three ideal landscape manager types may affect decisions about biodiversity-promoting activities (Christoffersen & Randrup 2024). The ideal types, *steward, master* and *facilitator* are intentionally generalised and may thus indeed all exist at once. Nevertheless, these are tendencies, with one usually predominating in any individual.

#### The Steward: Conserving Biodiversity as it Exists

Within this view of nature, biodiversity is a natural heritage that must be cared for by humans. Threatened species are of high concern, as are invasive species. Nature consists of measurable components (Vejre 2004), indicators assess these and prevail in arguments. In landscape management, focus is on elements such as dead wood, the giant tree, the meadow grass or the amphibian pond, but also the rare landscape type. Ethical arguments concern human intervention to protect natural and cultural heritage that in the stewards’ opinion are closely related (Barthel et al. 2005). The iconic landscape, including a diversity of native species, is highly appreciated.

#### The Master: Using Biodiversity for Services

Within this view of nature, biodiversity primarily provides functions for humans. This includes direct benefits such as timber, food and medicine, but also services that are more subtle and indirect (Windt et al. 2007). Economic valuation of ecosystem services thus represents a relatively recent way of commodifying nature, i.e. adapting species and ecological systems efficiently to contemporary human uses and market mechanisms (Gómez-Baggethun & Ruiz-Pérez 2011). Aesthetically, ‘modern’ utilisation of the landscape is appreciated^2^. Ethically, the continued supply of natural resources and services for the benefit of man in a long-term perspective, aka sustainable development, stands strong.

#### The Facilitator: Allowing Natural Processes

Within this view of nature, nature is self-sustaining and evolutionary, and the main management task is to enable a self-operating ecosystem. Species are more valued for their place and function in the system than for their placement on the redlist. Biodiversity will thrive when there is a continuity of trees, wetland, mycorrhiza and disturbance in the landscape, where habitats will move, disappear and re-emerge. Human companionship with non-human species facilitates these natural dynamics through prompting of disturbances. When continuity has been disrupted, natural processes can be advanced actively by e.g. veteranising trees, or passively by e.g. reducing care and maintenance.

## Methodology

### Selection of Respondents and Response Rate

We used the ‘local nature conservation’ effort, LONA, to identify the respondents. LONA is a Swedish national grant that aims to stimulate the long-term nature conservation commitment of municipalities in cooperation with non-profit associations^3^. Only three municipalities out of Sweden’s 290, have never had LONA grants. Using LONA-connected staff as respondents ensured a local involvement with nature conservation or restoration at some point, if not still. From the results we see that almost 40% were municipality-ecologists, but also forest managers, environmental strategists and managing directors were frequent respondents. We distributed the questionnaire, provided by *Netigate*, to two named landscape managers within each municipality, in a few cases only to one. In total, 500 potential respondents received the survey. The questionnaire was distributed via e-mail mid-February 2024, with a reminder sent out by the end of the month and a second in the beginning of March.

**Fig. 1:**
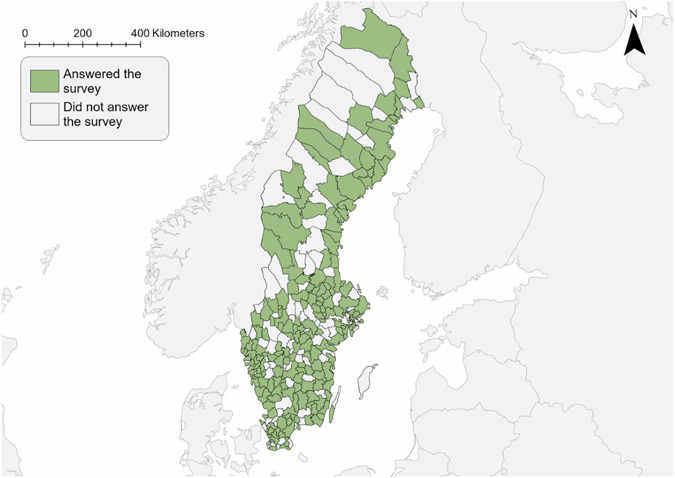
Distribution of the municipalities who were represented in the survey (map: Agnes Pierre)

We received answers from 274 local government landscape managers, representing 203 of 290 municipalities. This equals 70% of all Swedish municipalities, covering all parts of the terrestrial area (Fig. 1). The results are based on the 274 answers of the individual managers, which illuminate their perceptions or best knowledge. It was possible to share the questionnaire, i.e. two or more managers from the same municipalities may potentially have given different responses. Not all respondents completed the survey or answered all the open questions, which is why the number (n) may differ in the overview of the results.

### Elaboration and Design of the Survey

Prior to the survey, we performed three on site interviews with four public landscape managers, foremost ecologists, across Sweden. The objective was to know more about municipality activities related to nature protection, forest management and biodiversity accommodation broadly and ‘wilder’ initiatives, such as year-round grazing, reintroduction of fauna, restoration of natural hydrology, veteranisation of trees or forest burning, specifically. We asked questions about the political and administrative organisation as well. Insights gained from a Swedish national biodiversity conference, a webinar on biodiversity in practice and a public meeting on rewilding offered valuable practice-based perspectives to supplement the academic literature. Three biodiversity consultants helped formulate the questionnaire, providing input that shaped the questions addressing RQ1 and RQ2.

To identify potential trends in the predominant types of biodiversity measures implemented, we collected information on activities using matrices structured by distinct time periods. The design allowed for the clustering of answers in passive and active biodiversity accommodation, including ‘wilder’ activities, understood as activities that require action to instigate natural dynamics such as mentioned in the former paragraph, including for example the veteranisation of trees. To explore how biodiversity is prioritised within the organisation, we used multiple choice, and to identify potential other *public* partners we listed nine options and allowed for open additions.

Relating to RQ3 about the fundamental views of nature among public landscape managers, the views-of-nature typology (Christoffersen & Randrup 2024) allowed us to construct three ranking exercises relating to ethics, aesthetics and knowledge-based statements typical for each of the three ideal types of landscape managers that we presented formerly, *the steward, the master and the facilitator*. The ranking exercises aimed to determine if any of the ideal types prevailed among the municipal managers.

Furthermore, besides background information we asked about the respondents personal opinion, e.g. how they think their organisation ought to handle biodiversity in the future. We also asked them to write up to five words that they believe will be future keywords within management for biodiversity.

A pilot test of the survey was performed with the assistance of three municipal managers, different from the initially interviewed. Altogether, we had a total of 37 questions, with 15 opportunities to give open answers (see questions and results in Appendix 1).

### Data Analysis

We had 170 open answers to the question of what the future key concepts regarding biodiversity management will be, in sum 535 different terms and concepts. Initially we counted identical terms and concepts given by the landscape managers, then clustered similar terms and concepts into 83 broad themes that emerged from the data. Subsequently, we were able to further reduce the number of themes to 32. Half of them, the top 16 themes included 480 terms and concepts or 90% of them all. Table 1 shows how we grouped these.

**Table 1:** The synthesised results of the open question about future biodiversity management, the top 16 key themes

Rank	No.	Theme	Concepts and terms in the open answers
1.	90	Instrumental value for humans	Ecosystem service; Food purchases; Pollinators; Accessibility; Recreation; Public right; Carbon balance; Climate adaptation; Resistance to change; Functions; Systems; Nature tourism; Ecotourism; Leisure; Nature-based solutions; Multifunctionality; Synergy effects; Incorporation with other uses; Multifunctionality for producers; Usability; Part of municipal resources; Make use of; Biological=economic sustainability; Sustainability; Efficiency; Resource efficiency; Climate; Climate strategy; Resources; Agricultural land; Active agriculture
2.	52	Landscape level and scale	Landscape ecology; Differentiate landscape; Restore biotopes; Habitats; Restoration; Variation; Restore; Landscape perspective; Restore nature; Ecosystem; Landscape building; Diversify everywhere; Regeneration; Landscape management; Reservation; Enhance variation in the landscape; Reserve areas; Reserve at least 30%; Reclaim; Landscape planning; Natural ecosystem; Biological restoration; Land-use; Conservation; Balance land-use
3.	49	Knowledge	Knowledge dissemination; Insight; Education; Information; Pedagogy; Species knowledge; Experience sharing; Understanding; Competence; Educate; Skills development; Knowledge; Inventory; Nature value inventory; Research
4.	44	Protection	Formal protection; Protect; Increased protection; Protect most valuable; Species protection law; Protect what we have; Protected nature; Effective protection; Law; Protect from exploitation; Save what can be saved; Different species; Species protection; Many species; Stop species decline; Protect what is left
5.	38	Cooperation	Cooperation (inside + outside organisation); Cross-sectional cooperation; Network; Coordinate; Exchange of ideas; Collaboration; Common goals; Leave silo-thinking; Cross-border; Overall coordination; Collaboration between professions; Collaboration between dif. societal actors; Involvement; Engagement; All can do something; Citizen engagement; Participation; Flexible borders
6.	33	Nature as ‘relational’	(To) value; Interest; Nature value; Holistic view; Wholeness; Public interest; Societal interest; Life quality; Nature as everyday space; Ecosystem-based thinking; Nature near; Use without exhaust; children’s perspective; Caution; Biological/social value; Social dimension; Biological/ethical causes; Welfare; Health; Social-ecology; New thinking; Wonder; Proudness
7.	26	Active promotion	Rewilding; Freedom from interference; Transformative change; Creation; Create new; Create; Forward/push, Reintroduction; Nature development and grazing; Recreate; Natural grazing; Regeneration; Enlarge; Expand; Natural; Semi-natural; Cycle; Fresh nature; Action
8.	25	Green as infrastructure	Green infrastructure; Link together; Connectivity; Transition zones; Connection; Green structure; Green-blue (infrastructure); Green-blue planning; Urban; Within urban development; Urban green structure; More urban nature
9.	23	Economy-dependent	Economically sustainable management; Funding; Economy; Economic situation; Economic causes; Land-owner economy; Compensation; International Aid Services
10.	19	(Political) management	Political management; Management plan; Strategy; Sustainable management; Adaptive system-aligned management; National guidelines; Planning; Ecosystem-based decisions; Management; Forest politics; Politician engagement; Managers; Land management; Anchoring; Political anchoring
11.	17	Forestry	Continuous cover forestry; Stop clear cut; Nature-near forestry; Forest; Stratified old-growth forest; Old Forest; Trees; Deciduous trees; Leaf volume
12.	17	Blaming, human induced causes	Development; Societal development; Climate damage; Climate change effects; Littering in sea; Exploitation; Consumption; Over-population; Pesticide; Mass extinction; Habitat loss; Extinction dept; Consumed; Stop urban densification; Zero profit interests
13.	15	Human care	Nurture; Care; Steward; Differentiated care; Care for lawns and parks; Maintenance measures; Consider; Proper care; Concern; Adapted care; Responsibility; Advocacy; Biological cultural heritage
14.	12	Water related	Wetland; Rewet; Watercourses; Limnic restoration; Restore wetlands
15.	10	Priority & Urgency	Urgency; Priority; Prioritise; ‘Everything everywhere all at once'
16.	10	Long term effort	Long term; Continuity; Future proof; Long-term decisions; Long term sustainability; Ecological long-termism; Political understanding of long-term benefit

No. refers to the times the concept or term was mentioned. The top 16 key themes include 90% of terms and concepts in the answers

In analysing the three ranking exercises intended to reveal each landscape manager’s dominant view of nature, placement within an ideal type was determined by two ‘highest-ranked’ arguments associated with either the *steward, master* or *facilitator* categories. Of 201 respondents, 14 were uncategorisable and excluded from further calculations.

## Results

### Biodiversity Accommodating Measures

#### Landscape Level

**Fig. 2:**
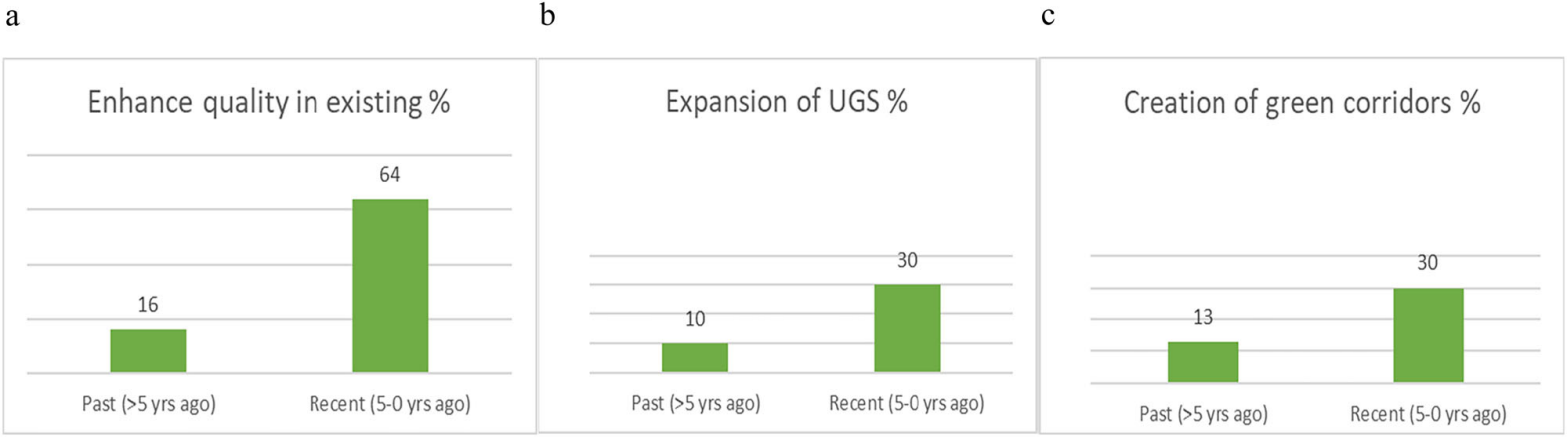
Increasing tendencies within the management for biodiversity at landscape level (in % of answers) as perceived by managers. **a** Quality of biodiversity in existing green spaces, **b** amount of green spaces and **c** creation of green corridors

We detected clear increasing trends (as perceived by the respondents), illustrated in Fig. 2a, b and c. Enhancing the biological quality in existing areas has become a common measure, 64% of the respondents answered they had done so within the municipality in the recent past. Likewise, 30% of all respondents answered that they experience an expansion of green areas in the city. Another positive trend to date is the creation of green corridors.

We also found a clear decreasing trend relating to strategic initiatives for biodiversity at landscape level. In the past, nature reserves were established as a main measure, but this has decreased. Similar trends are found for the certification of forests and to some degree in relation to taking forest out of production.

The 124 open answers relating to efforts at landscape level foremost related to the establishment or restauration of water bodies, such as wetlands and watercourses, followed by the performance of inventories. Also support of specific species and landscape-types were mentioned.

#### Local Places

**Fig. 3:**
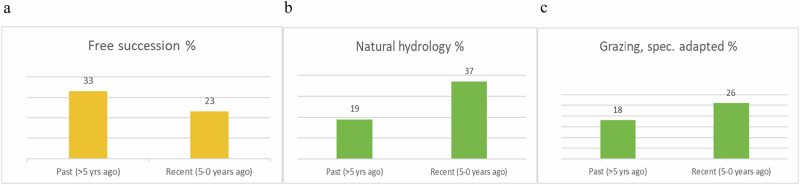
Tendencies within the management for biodiversity at local level as perceived by managers. Presented as % of answers in (**a**) free succession of the vegetation, **b** natural hydrology and **c** grazing, specifically adapted to nature conservation

A trend away from the free succession of vegetation was a tendency in ‘Strategic measures to promote biological diversity at the site’ (Fig. 3a) and only 4% of respondents foresaw this as a measure in the near future^4^. The increasing trends go towards the reestablishment of natural hydrology, and grazing that is specifically adapted to nature conservation, such as the use of tree browsers (Fig. 3b and c). Larger disturbances such as forest-burning or fauna-reintroduction, however, are ‘not discussed’ (44% and 40% respectively). There seems to be an interest though since 21/18% answer ‘perhaps in the future’.

Also this topic generated many open answers, 73 altogether. They clustered around rewetting, but also the active promotion of habitats primarily for insects and birds through meadow development, the veteranisation of trees, leaving dead wood (both standing and lying) and fauna depots. Soil disturbances and purposeful thinning for variety and structure in urban forests were mentioned too by several managers.

#### Forests

**Fig. 4:**
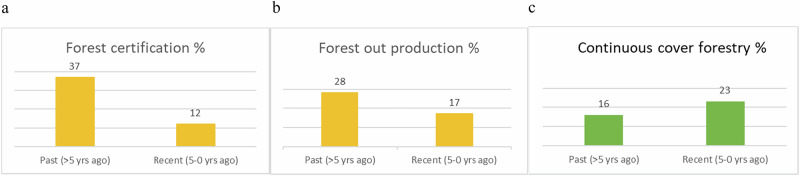
**a**–**c** Tendencies within municipality forest management as perceived by managers (in % of all answers) in **a** certification of production forest, **b** forests taken out of production and **c** CCF

As regards forest management, the certification of production forest to brand a certain level of sustainability was a common acquirement in the past, as was taking forest out of production, but both measures have decreased in occurrences (Fig. 4a, b). Instead, continuous cover forestry (CCF) seems to be on the increase (Fig. 4c). It is also foreseen to happen in the future, 12% answer they will start over the coming five years, while 17% say perhaps further ahead.

### Biodiversity Within the Municipal Organization

#### Prioritisation and Financing

**Fig. 5:**
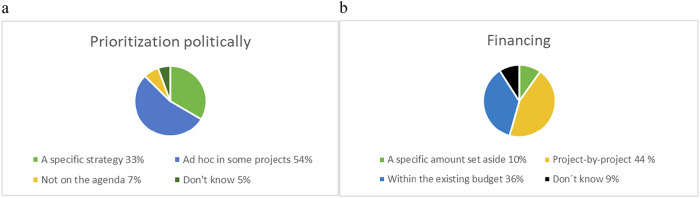
**a**–**b** Prioritisation **a** and funding **b** of biodiversity measures in municipalities in %

We asked how biodiversity is prioritised politically and how measures are financed in each municipality. The answers were distributed as seen in Fig. 5a, b:

Biodiversity accommodation is primarily applied ad hoc in some projects and financed project-by-project. Contrary to this, one third of the respondents stated that their municipality has a specific strategy to address biodiversity, which is however not followed by a corresponding amount of financial resources set-aside. Most funds for biodiversity promotion activities are found within the existing budget.

Responsibilities for biodiversity within the municipal organization are primarily transversal (36%). Only 19% answered that responsibility for biodiversity accommodation rests within a specific department.

Half of the municipally owned forests are managed by the municipality itself, while every fourth municipality (26%) has their forest managed by an external company. The open answers confirmed this but indicate that a mixed arrangement is prevalent, as the most urban-near forests may be managed by the urban landscape manager, while the more remote forests may be managed by a municipal or county ecologist or forester, or outsourced to a private forest company.

We had 26 open answers about how the municipal organisation ought to manage biodiversity in the future. Some answers included multiple suggestions. Political priority was mentioned the most, often connected to planning and funding anchored herein. Financial resources were stressed as a prerequisite for working seriously for biodiversity, not only in the form of funding from national level, but as a priority at local level:

*Although I work in a municipality with good finances, projects related to nature conservation are dependent on government funding through various grants*

Knowledge was also a recurring theme, and it concerned knowledge within the municipality organisation itself. Only once was knowledge about biodiversity in society at large mentioned. Dialog with land and water owners appeared several times as necessary, including also the need of restrictions towards certain land use activities. Finally, the municipal organisations’ own land use practices were highlighted, e.g. ‘unconcern’ with natural land:

*In a few decades we exploit several hundred hectares of nature (irreversible erasure of diverse natural land). This is possible if there is no short list of strictly protected species. Parrying provides marginal “compensation”*

In a closed answer question to the same (how the municipality ought to manage?) with the opportunity to choose several answers, the focus was on the ‘protection of valuable landscape types and elements’ (94%), followed by the ‘necessity of biodiversity included in multifunctional ecosystems’ (90%) and ‘transversal collaboration’ (83%). Also ‘collaboration with new actors had a high score (75%).

#### Co-management Between Public Institutions

Efforts to accommodate biodiversity are rarely done by the single municipality alone. Only 11% answered they accommodated biodiversity without cooperating with other public institutions. Working in cooperation with other municipalities and/or the county administration was the most common answer, 44% (*n* = 121) confirmed this partnership. Cooperation with the Forest Agency was the second most common answer with 27% (*n* = 73). Out of 188 open responses to the same question, 56 singled out the partnership with the county administration.

### Tendencies in Views of Nature

**Fig. 6:**
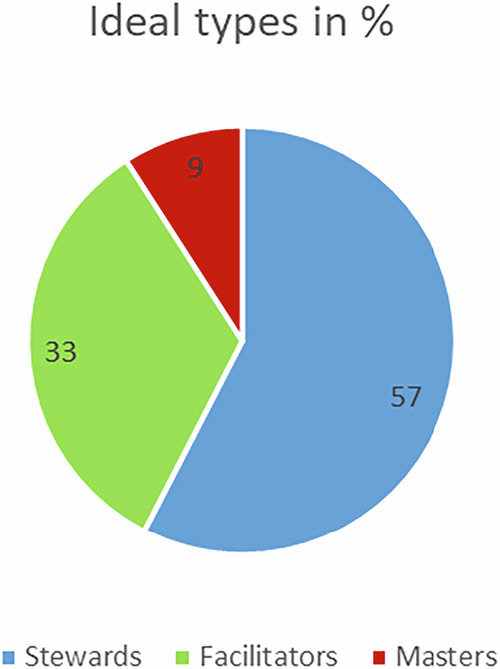
Dominating views of nature among managers

The three ranking exercises pointed to the following distribution of municipal landscape managers within the views-of-nature typology (Fig. 6).

**Fig. 7:**
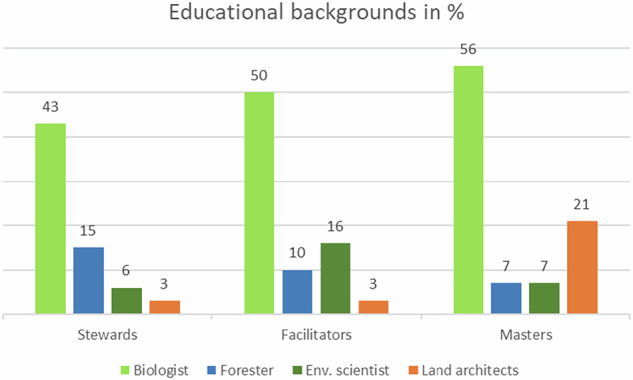
Educational backgrounds of the three ideal types

Most managers have biology as educational background. However, the second most common educational background differs depending on the view of nature. Respondents with master view commonly have a degree in landscape architecture, while the facilitators’ second most common degree is within environmental education (Fig. 7).

The *masters* are the youngest with a median age of 38 years compared to *stewards* and *facilitators* with 49 and 46 years, respectively.

**Fig. 8:**
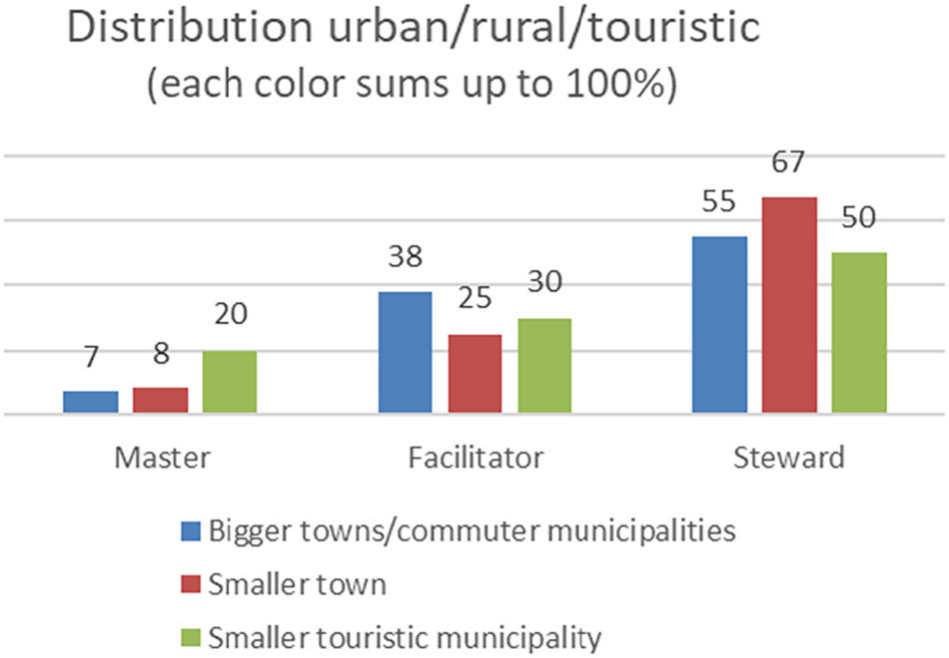
Predominant workplace of the ideal type of landscape manager. Each colour sums up to 100%

Masters are also, by percentage, employed in smaller municipalities with tourism (57%), while facilitators are proportionally mostly employed in the bigger towns/commuter municipalities. Stewards are mostly working in the smaller towns (Fig. 8)^5^.

### Keywords Denoting the Future of Biodiversity Management

Table 1 shows how we grouped the large number of open answers regarding future key concepts in biodiversity management.

## Discussion

Together the answers to the research questions help us understand the current state and trends in landscape management for biodiversity across Swedish municipalities, as perceived by managers, and whether measures are taking a wilder direction.

### Carefully Wilder Management

We understand the high focus on improving natural qualities of existing urban green spaces as a growing acceptance of changes in aesthetics, in the population (Unterweger et al. 2017) as well as among managers, most probably emerging from an increasing acknowledgement of the importance of urban biodiversity accommodation. Biodiversity-supporting landscapes look ‘messier’ than the conventionally managed landscape, promoting ecological value over meticulous tidiness, and landscape managers are concerned with the public appraisal of their practice (Brocki et al. 2025, Hoyle et al. 2017). The conversion from mown lawn to meadow, or just long grass, appears to be a common approach in the inner urban areas, as well as dead wood and other organic material left behind. It may simultaneously reflect the fact that it is difficult to acquire new land for this purpose (Haaland and Konijnendijk 2015), yet we found a perception of an increasing trend in the expansion of urban green areas, as well as in green corridors. This increase in new green areas and green corridors may appear surprising considering a wide perception of decreasing urban green areas due to e.g. densification (ibid). A recent Nordic study, based on interviews with municipal green space managers, explained that densification was perceived as a common threat to urban green spaces independently of municipal size, history and context. However, there was also a managerial perception that urban densification is leading to more spaces to be managed, the reason being that most densification processes are located on derelict land (previous harbour and transport areas), not previously formally managed (Randrup et al. 2021). It remains unclear whether the same holds true for this study; however, this may account for the somewhat unexpected perception that urban green areas have expanded during the past five years.

When looking at activities that improve biological qualities at municipal land overall, we can detect a move from ‘passive rewilding’, i.e. from the free succession of vegetation, towards instigating disturbances to accelerate natural processes through restorations of natural hydrology and grazing specifically adapted to nature conservation. The use of grazing and browsing animals, i.e. the role of non-human species in new landscape ‘engineering’ and management, is considered important for landscape dynamics. More than just management tools, the animals, in partnership with humans, become an integrated part of the eco-system (Prior & Ward 2016; Lorimer 2015; Baerselman & Vera 1995). Likewise, the restoration of natural hydrology is a central instrument to create habitats for other life forms (Torres et al. 2018; Svenning et al. 2016). On the other hand, we rarely saw anyone answering that they applied larger disturbances, such as forest fire or the reintroduction of fauna. Our interpretation is that management of urban green spaces has become somewhat, but only carefully, wilder.

Forestry has a significant cultural history in Sweden (see e.g. Lundmark 2020). Earnings from production has traditionally contributed to municipal economies but since the latest decade of the former century, nature conservation has become increasingly important (Rydberg & Falck 2000). How to promote ‘nature’ has, however, changed over time. Our results show that in comparison to past dominating practices, where forests were either certified production or, near urban areas, taken out of production, experiments with continuous cover forestry (CCF) is predicted by the respondents to take place in municipalities in the future. This reflects and follows the trend in and demand from the EU for the forestry sector in general^6^, and Sweden specifically (Pokorny-Kindlman 2024). There is now a turn towards more nature-near management, which throughout Europe has resulted in established CCF systems but which in Sweden has met some resistance (Hertog et al. 2022). Urban and municipal forests, however, have a history that for half a century has pursued goals linked to human recreation and enjoyment, and thus appear more diverse in structure and composition than the average production forest (Rydberg & Falck 2000). It is probable that municipal forests will be frontrunners regarding CCF given their longstanding consideration for forests’ multifunctionality.

While fulfilling a range of societal needs, CCF may however not be the panacea that promotors claim (Bürgi 2015) and its benefit for biodiversity still depends on the intensity of production (Blattert et al. 2023) and specifics of management (Dafis 2001). A Danish study shows varied results for biodiversity due to the darker forests that a dense cover in the crown promote (Klynge et al. 2020). Biodiversity is accommodated by disturbances that create light and dead wood of which a broad range of plants, animals and fungi depend at some point of their life cycle (Swanson et al. 2014; Hilmers et al. 2018). Open answers of our survey indicate that many landscape and urban forest managers increasingly are aware of that.

### Ad hoc Organisation and Outsourced Forest Management

Biodiversity measures in projects and general management are primarily treated ad hoc and financed from case to case, often within existing budgets. Responsibility to ensure the accommodation of biodiversity is also somewhat unclear, i.e. transversal and/or without specific people designated. Many landscape managers thus appear to include dedicated partners outside of the organisation to promote specific biodiversity promoting tasks.

Elander and colleagues (2005) found in their study of four cities in Sweden that in local plans, the concept of biodiversity was used in a very general way and hardly mentioned at all unless there was a specific protection interest in the area. No clear guidelines were provided, so the task of translating the general statements into action was left to the implementing manager. We must visit global or other European studies to find newer results regarding biodiversity policies. Runhaar et al. (2024) find that on a global level, biodiversity policy integration, i.e. how biodiversity targets are being mainstreamed into sectoral policies, generally remain low and abstract. They argue for a shift from voluntary towards mandatory responsibilities. In a Swiss study covering the first two decades of the 21^st^ century, Reber et al. (2022) found that biodiversity policy integration is rarely sustained. Its attention increases temporarily, reflecting political cycles. These findings align well with answers in our preliminary interviews. It suggests that without specific targeting, the overall ambition of always considering biodiversity in the green space management or urban forestry depends on the single landscape or forest manager, and thus to some degree on his or her personal interest or fundamental view of nature. Singh et al. (2021) label this phenomenon a lack of vertical alignment within the public organisation.

It appears that parts of the municipally owned forests are managed conventionally with the purpose of contributing to the municipal economy, conditions which were confirmed by two landscape engineers from Umeå municipality in a preliminary interview. Here, about 1000 ha is managed as forested nature with recreational and biodiversity purposes, while 10 times as much is subject to the demand of production. They explained that a detail-planned forest can obtain dispensation from the Swedish forest law [to be taken out of production]. On the downside, they added, a detail-planned forest can ‘lock out’ a natural development of an area. Nevertheless, municipal landscape managers have a good foundation to plan for wilder and more biodiverse forest areas in long term strategic planning, either by reclaiming management, or setting new guidelines for entrepreneurs. While important information about privately owned forested lands is inaccessible for municipal landscape managers (Stjernström et al. 2018; Thellbro et al. 2017), own land – i.e. public commons – seems to constitute a valuable, ‘low-hanging’ opportunity for public biodiversity accommodation.

### Stewards Dominate Landscape Management

Not surprisingly, *stewards* constituted the dominating landscape manager ideal type within the municipalities. According to Christoffersen & Randrup (2024), the steward argues that species, populations and habitats need continuation of appropriate and professional human management to prevent their extinction. The steward tends to divide the landscape into separate elements or landscape types that each demand certain maintenance and thus is well aligned with the municipal organisational framework. Quitzau et al. (2022) identify and describe how organisational ‘silos’ affect implementation of sustainable-development goals in Nordic municipalities. Singleton (2023:7) discusses this too, arguing that *“As befits a hierarchical organisation, municipalities organise knowledge and expertise by dividing it up into areas of specialisation and expertise”*, and found that even when there is an articulated desire for more holistic interpretations of biodiversity in urban planning and management, biodiversity mostly emerges as “*a hierarchical view of reality”* (ibid:1), i.e. as a measurable object, an indicator or a characteristic.

Perhaps the municipal organisation is more decisive for management practices than the profession of the manager. Still, there was an interesting pattern when looking at professions in second place within the three ideal types. The facilitator had an environmental educational background, i.e. has studied issues related to how natural systems work and how to manage or protect natural resources, allowing for an understanding of the importance of promoting natural dynamics to restore or create ecological processes by e.g. seeing landscapes as self-adjusting and evolutionary ecosystems (Christoffersen & Randrup 2024). The *master* had landscape architects in second place. The master may well acknowledge the dynamics of the ecosystem but focus on its services to man; the human control of the processes is essential to make the city more resilient. Biodiversity is a potential added value or benefit when green infrastructure must solve multiple challenges. The design element of landscape architecture aligns well with this approach.

If our survey reveals only a careful movement towards facilitation of natural processes, the ranking exercises indicate a (surprisingly) high percentage of landscape managers sympathising with this view. We can however identify a clash with the organisational everyday practice that delimit its expansion (e.g. Sang et al. 2021). One thing is the hierarchical logic of planning and maintenance, another the several other considerations the landscape manager must observe and address in an urban setting e.g. storm water management (Qiao et al. 2018), or public health (Sunding et al. 2024). While the *facilitator* potentially may instigate a new view of nature and a different approach to management, its implementation does not seem easy, nor likely. The ideas about a wilder landscape appear to thrive relatively better in the urban areas, since this is where facilitators (relatively) occupy most positions. An urban-rural divide, as can be an interpretation of the results, may be explained by the urban physical and psychological distance to daily occupation with nature as an economic resource, and the rural as a place for a lifestyle considered “stable” by the rural population (Peeren 2019). Gabehart (2025) also discusses conservation as regards wolf management and argue that for rural communities, conflictual views of conservation may touch upon matters of place-based identity, while for urbanities, environmental issues should just be practically dealt with.

Mastery may constitute a new frame for municipal landscape management. While the trend appears to be modest, the masters form the youngest group of managers, and their ideas align well with today’s discourse of multifunctionality of green spaces to address several purposes (Sang et al. 2021), which is also supported by recent policy developments. However, von Post et al. (2023) show how the Swedish green infrastructure policy assemblage, despite a current focus on biodiversity, increasingly is formed by a focus on land uses for purposes other than conservation. Albeit there is a potential to mainstream biodiversity accommodating measures across different sectors, they find that policies will likely promote green infrastructures that do not disrupt existent activities, which delimit the capacity to halt biodiversity loss (ibid). That masters predominantly work in the touristic areas make good sense; they have an incentive to apply multiple functions of green spaces, e.g. delimit productional areas, or at least adapt them to recreational purposes, that in these areas represent also economic values.

Views of nature were addressed by testing a typology (Christoffersen and Randrup 2024) to see how its hypothesis relates to reality. The results confirm the possibility of dividing landscape managers into the identified ideal types, and they added to the typology by inflicting aspects related to e.g. age, education and placement along a rural-urban gradient. We are aware that the ideal types are not ‘clean’ but rather describe tendencies.

Contemporary practices and organisational structures may reproduce a dominant view of nature, rather than embrace innovation towards radically different management practices. Institutional contexts, regulations and policies all affect which view dominates, but as we saw, also funding or knowledge has impact. Knowing that, on top of this, many different factors influence decisions on how to implement biodiversity accommodation (Dawson et al. 2017), we do not discuss trends in the survey that may indicate patterns with regards to fundamental underlying views of nature of the landscape managers and the accommodation of biodiversity implemented in his or her municipality.

### Future Management for Biodiversity

The key concepts given by the landscape managers in an open answer help us project future biodiversity management. The answers may reflect expectation, fear, wishful thinking, a plan or a belief, so we cannot link them to the ideal type of each respondent. However, the characteristics of the different views of nature are useful here. In the following we address only some of the highest-ranking clusters of terms and concepts (Table 1).

*Instrumental value for humans* gathered far the most (almost 20%) of the key terms and concepts. In academia, ecosystem services and spatial multifunctionality has been growing fields of research for decades (e.g. Garland at al. 2021; Hölting et al. 2019). The key concepts suggested by the practitioners establish that this is also a municipal planning and management reality, although only a minority of the managers share this ‘master-view’. More tasks need to be solved within the existing green areas (Sang et al. 2021; Windt et al. 2007) and meet socio-economic goals (Gómez-Baggethun & Ruiz-Pérez, 2013). Biodiversity accommodation is just one; whether it will compromise remains to be seen.

Second ranks the importance of addressing biodiversity at a landscape or habitat level. This is in line with, not only the trends we saw in this survey, but international attention to the importance of shifting the focus from species-specific efforts towards more holistic landscape-scale strategies that integrate patches and corridors to support species mobility and ecosystem functioning (Babu 2023).

Third ranks concepts related to *Knowledge*. Learning, knowing, disseminating, researching and educating about biodiversity will be an important, ongoing task of the landscape manager, perhaps establishing the fact of the steward that the responsibility is foremost with the professional (Christoffersen & Randrup 2024). Further, the steward’s opinion that biodiversity needs *Protection*, is clearly seen in fourth place. Formal, preferably legal, protection of what is left of nature or threatened among species and habitats (Vejre 2004) must still apply in the future. *Protection* was also predominating the answers to how the municipality ought to manage for biodiversity. The argument does not align well with the highest-ranking key concept (instrumental value for humans); we foresee increasing discussions and disputes regarding biodiversity concerns on the one hand, and human concerns on the other.

*Cooperation* ranks fifth and regards collaboration in a broad sense: across sectors, professions, knowledge-holders, social actor groups and citizens. The conviction that addressing the so-called wicked problem takes a wide range of inputs and inclusive governance structures (Head 2023) will stay prominent in future management for biodiversity. Lastly, we will mention *Active promotion* since this was central in our study: does management for biodiversity include the active promotion of wilder landscapes? We can establish that it exists among measures taken, and exists among future key concepts, but in neither case the concept is dominating in Swedish municipality management for biodiversity. While 1/3 identified as facilitators, this view and approach was only ranked 7^th^ as an anticipated future key concept.

### Methodological Reflections

Our respondents occupy different positions in the municipal organisations and have different responsibilities. Also, the survey has been shared, passed on and filled out in groups; we do not know exactly who the respondents are. Some have administrative or semi-political roles and may, when responding to questionnaires, be influenced by psychological aspects and contextual factors other than those related to the topic of biodiversity.

Role identity influence responses. Public managers may answer in ways that reflect their professional self-concept, such as being policy implementers or public-interest guardians, rather than personal views or perceptions (Van der Meer 2024). Social desirability bias is also a concern, as respondents tend to align their answers with perceived normative expectations or policy ideals, particularly on sensitive topics such as sustainability or biodiversity promotion (Bursztyn et al. 2025; Krumpal 2013). Fear of repercussions, even under anonymity assurances, can lead to cautious or vague answers, as concerns about reputational or institutional consequences persist (Tourangeau & Yan 2007). Additionally, strategic answering may occur when respondents seek to justify constraints or signal compliance with political agendas, creating a gap between reported intentions and actual practices.

These dynamics underscore the importance of interpreting survey data critically, acknowledging that responses may reflect desired behaviour signalling rather than authentic operational realities.

## Conclusive Remarks

With our survey, we set out to explore Swedish municipal landscape managers’ approaches to biodiversity promotion, and whether they reveal a ‘wilder’ trend. We found trends towards managing urban landscapes, including urban forests, in a carefully wilder manner. However, it also appeared that this was due to policy and stakeholder demands combined with the managers’ own initiative, rather than a clear organisational strategy. Municipally owned land, for instance forests, holds a large potential for the accommodation of biodiversity, but it takes political will and organisational strategy to plan and manage for that.

We also found a second, somehow competing trend, namely that of multifunctionality regarding ecosystem services in urban landscapes, reflecting the view of a master. While far the most landscape managers align with conservationist or nature facilitation values, when choosing keywords that describe future management for biodiversity, they pointed in that direction. Those few who responded as masters in our study also constituted a younger generation. An attentive projection of future biodiversity management thus confirms this trend, based on the political demands and physical conditions, i.e. limited green areas for the various merits of green spaces regarding benefits to human society.

A surprisingly large group of landscape managers, however, hold the view of nature of a facilitator, i.e. believe in the biodiversity accommodating values of a wilder landscape. Organisational constraints and overlapping demands, including factors not brought up in the survey, explained some of the barriers that prevent the management for such landscapes.

## Supplementary information

Supplementary information

## Data Availability

The result report from the survey provider is attached as a pdf file called Appendix 1.

## Footnotes

1 Biodiversity encompasses in its definition the genetic and species variety and the ecological complexes (i.e. habitats, dynamic landscapes and ecosystems) of which they are part.

2 The term ‘modern’ is employed here in opposition to ‘traditional’, indicating practices characterized by rationality and efficiency.

3 LONA – Lokala naturvårdssatsningen (‘The local nature management initiative’ – authors’ translation)

4 In our illustrations we only show what has already been carried out (past and present) according to the respondents’ best knowledge

5 Municipal group division according to *Sweden’s Municipalities and Regions* (SKR)

6 The New EU Forest Strategy for 2030; The European Commission Biodiversity Strategy for 2030: The EU Nature Restoration Regulation
