# Supplementary material for: Accommodation of Biodiversity in Swedish Municipalities. A National Survey
Source: Environ Manage. 2026 Apr 10;76(5):146. doi: 10.1007/s00267-026-02429-w (PMC13068711; doi:10.1007/s00267-026-02429-w)
Supplement: Supplementary file 1 — Supplementary information [file 267_2026_2429_MOESM1_ESM.pdf]

Biologisk Mångfald

Undersökningsinformation

197 / 491
SLUTFÖRDA

536
UTSKICK

92 %
SVARSFREKVENS

Hur prioriteras biologisk mångfald politiskt i din organisation?

Sida 1 - Fråga 1 | Om kommunens/länets strategi för biologisk mångfald

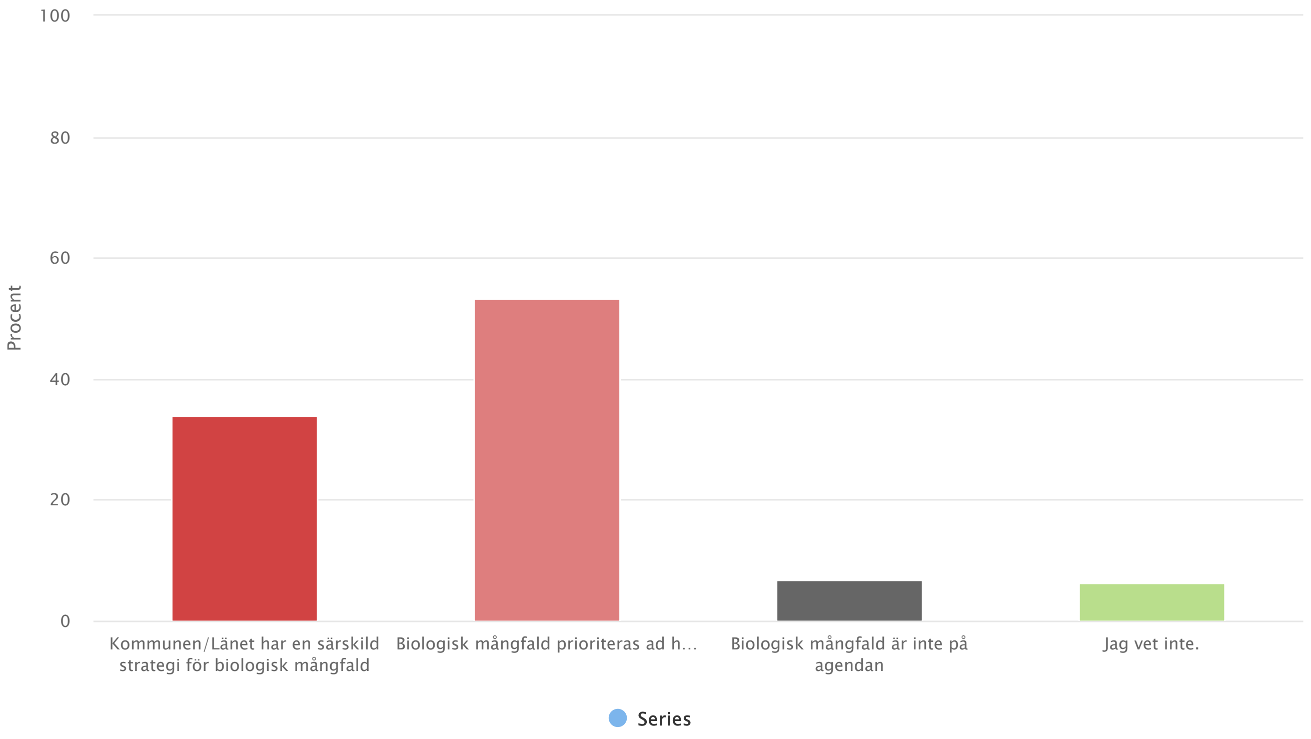

1	Kommunen/Länet har en särskild strategi för biologisk mångfald	96 (34%)
2	Biologisk mångfald prioriteras ad hoc i vissa projekt	151 (53%)
3	Biologisk mångfald är inte på agendan	19 (7%)
4	Jag vet inte.	18 (6%)
	Svar	284

Var ligger det organisatoriska ansvaret för biologisk mångfald i din organisation?

Sida 1 - Fråga 2 | Om kommunens/länets strategi för biologisk mångfald

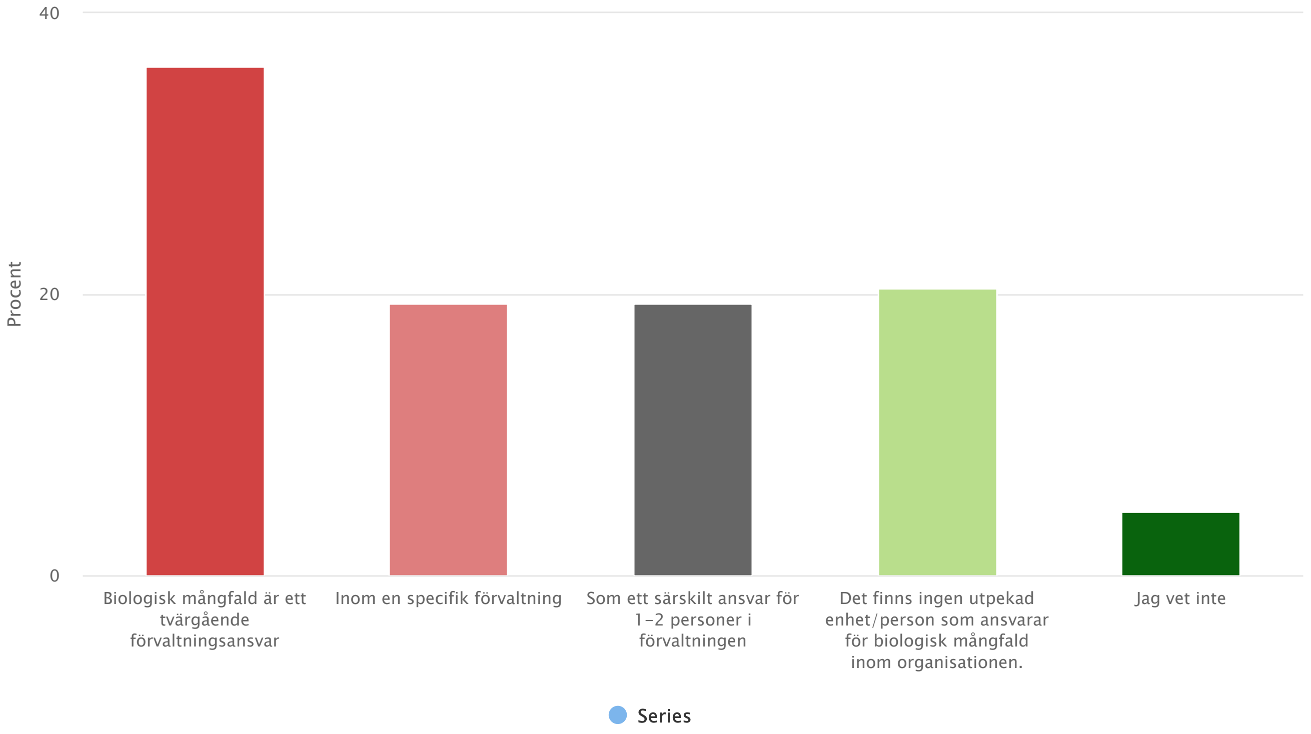

1	Biologisk mångfald är ett tvärgående förvaltningsansvar	103 (36%)
2	Inom en specifik förvaltning	55 (19%)
3	Som ett särskilt ansvar för 1-2 personer i förvaltningen	55 (19%)
4	Det finns ingen utpekad enhet/person som ansvarar för biologisk mångfald inom organisationen.	58 (20%)
5	Jag vet inte	13 (5%)
	Svar	284

Hur finansieras insatser för biologisk mångfald i din organisation?

Sida 1 - Fråga 3 | Om kommunens/länets strategi för biologisk mångfald

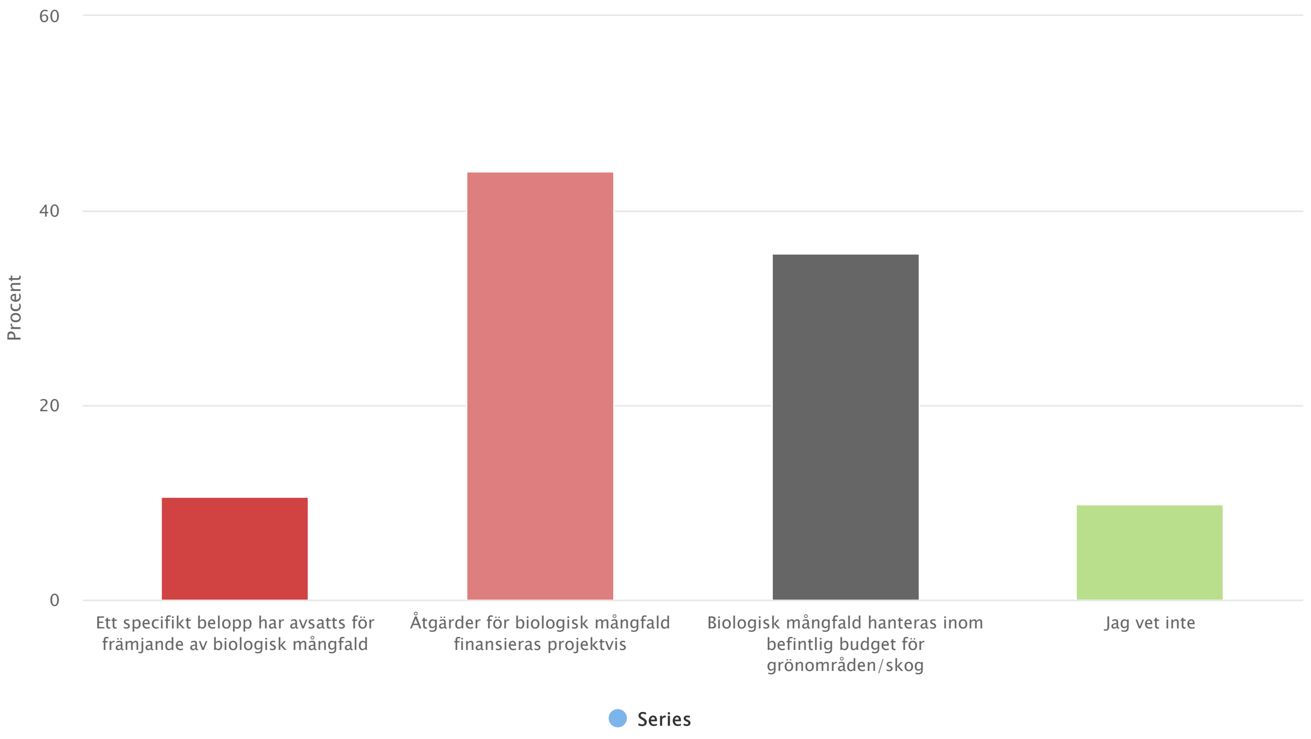

1 Ett specifikt belopp har avsatts för främjande av biologisk mångfald	30 (11%)
2 Åtgärder för biologisk mångfald finansieras projektvis	125 (44%)
3 Biologisk mångfald hanteras inom befintlig budget för grönområden/skog	101 (36%)
4 Jag vet inte	28 (10%)
Svar	284

I samarbete med vilka andra offentliga eller halvoffentliga organisationer/företag sker insatser för biologisk mångfald? Flera kryss kan sättas.

Sida 1 - Fråga 4 | Om kommunens/länets strategi för biologisk mångfald

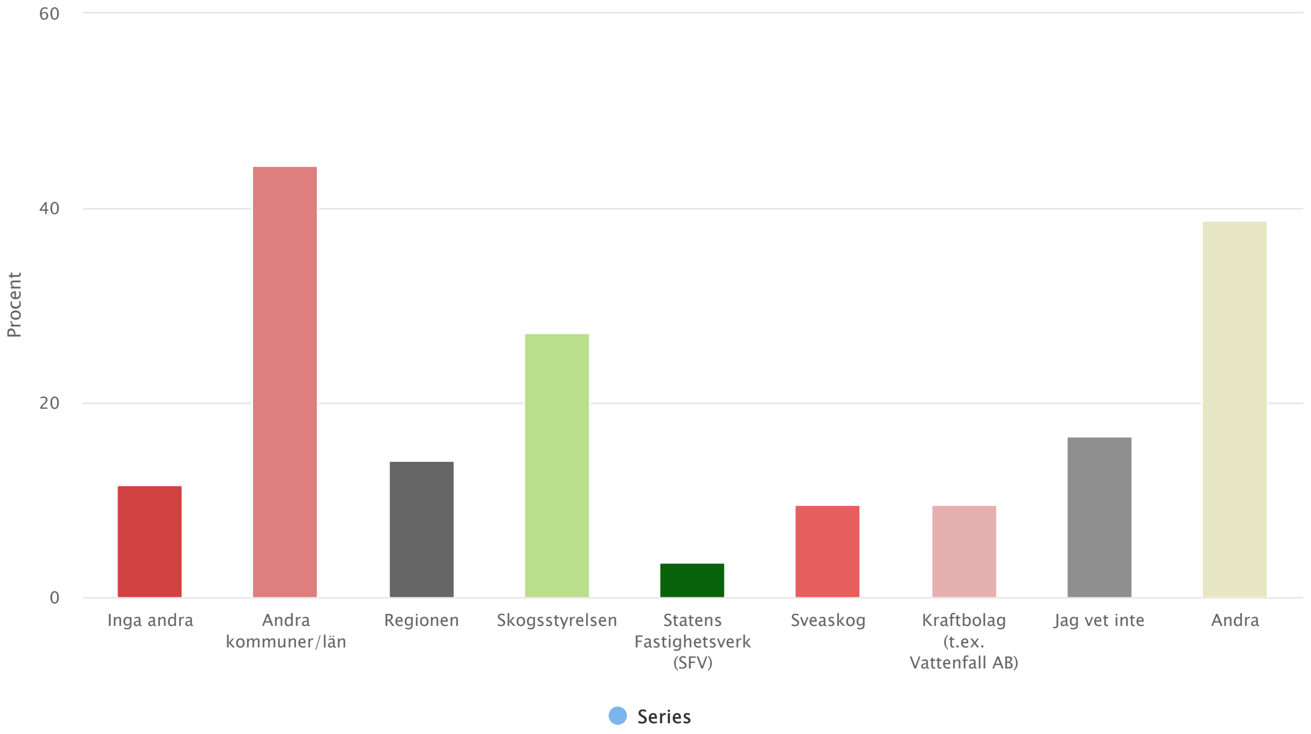

1 Inga andra	33 (12%)
2 Andra kommuner/län	126 (44%)
3 Regionen	40 (14%)
4 Skogsstyrelsen	77 (27%)
5 Statens Fastighetsverk (SFV)	10 (4%)
6 Sveaskog	27 (10%)
7 Kraftbolag (t.ex. Vattenfall AB)	27 (10%)
8 Jag vet inte	47 (17%)
9 Andra	110 (39%)
Svar	284

Andra

Sida 1 - Fråga 5 | Om kommunens/länets strategi för biologisk mångfald

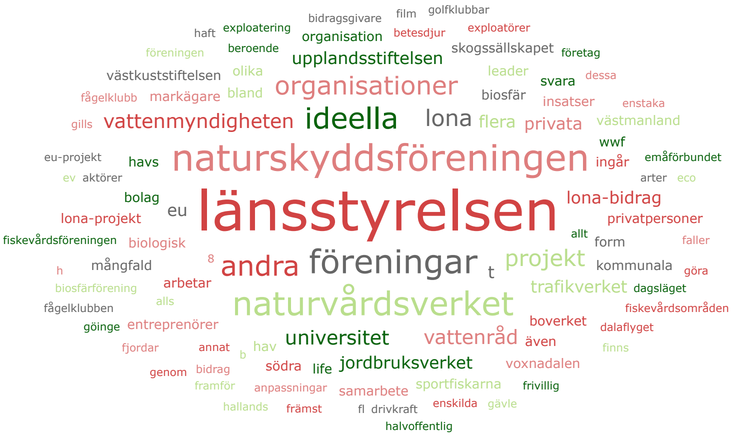

Kommun BGD

Sida 1 - Fråga 6 | Om kommunens/länets strategi för biologisk mångfald

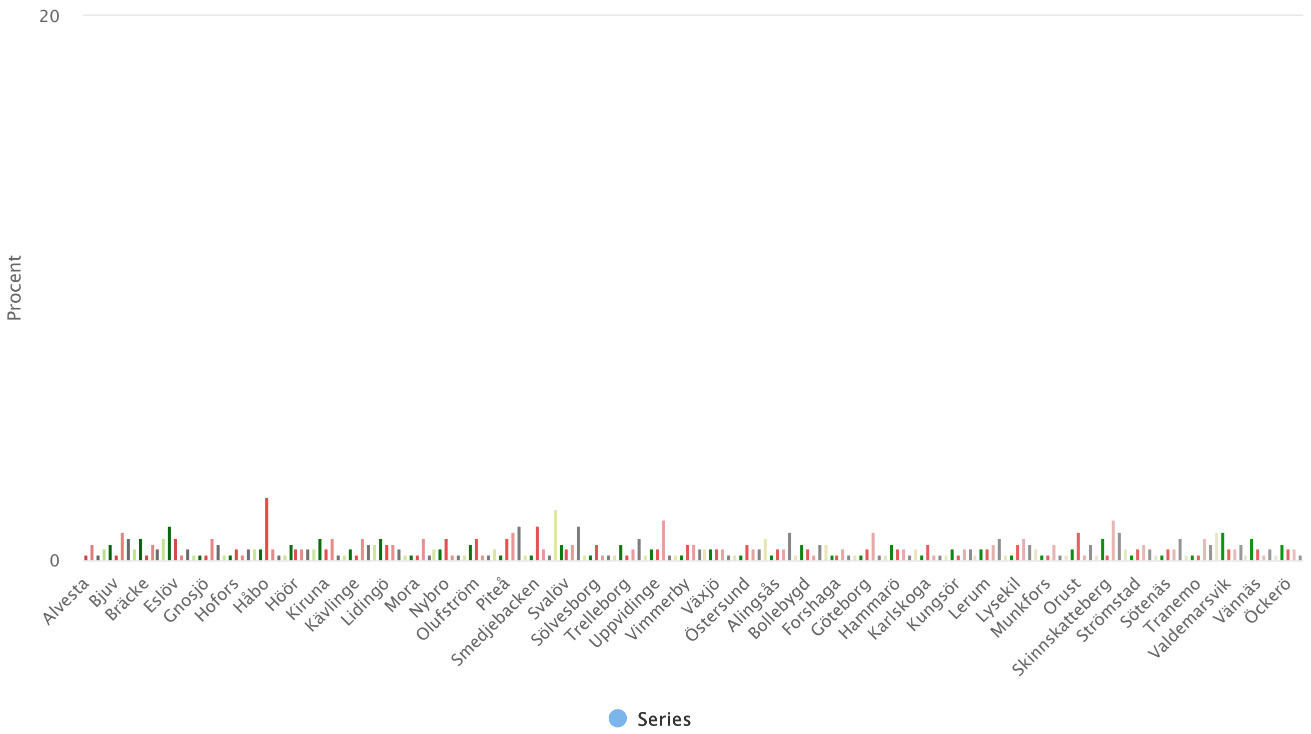

1 Alvesta	1 (0%)
2 Aneby	3 (1%)
3 Arvidsjaur	1 (0%)
4 Avesta	2 (0%)
5 Berg	3 (1%)
6 Bjuv	1 (0%)
7 Boden	5 (1%)
8 Bollnäs	4 (1%)
9 Borlänge	2 (0%)
10 Botkyrka	4 (1%)
11 Bräcke	1 (0%)
12 Båstad	3 (1%)
13 Ekerö	2 (0%)
14 Emmaboda	4 (1%)
15 Enköping	6 (1%)
16 Eslöv	4 (1%)
17 Falkenberg	1 (0%)
18 Falun	2 (0%)

[illegible]		[illegible]
19	Gislaved	1 (0%)
20	Gnesta	1 (0%)
21	Gnosjö	1 (0%)
22	Gävle	4 (1%)
23	Halmstad	3 (1%)
24	Haparanda	1 (0%)
25	Hedemora	1 (0%)
26	Hofors	2 (0%)
27	Huddinge	1 (0%)
28	Hudiksvall	2 (0%)
29	Hultsfred	2 (0%)
30	Hylte	2 (0%)
31	Håbo	11 (2%)
32	Härjedalen	2 (0%)
33	Hässleholm	1 (0%)
34	Höganäs	1 (0%)
35	Hörby	3 (1%)
36	Höör	2 (0%)
37	Järfälla	2 (0%)
38	Jönköping	2 (0%)
39	Kalmar	2 (0%)
40	Karlskrona	4 (1%)
41	Kiruna	2 (0%)
42	Klippan	4 (1%)
43	Kristianstad	1 (0%)
44	Krokom	1 (0%)
45	Kungsbacka	2 (0%)
46	Kävlinge	1 (0%)
47	Laholm	4 (1%)
48	Landskrona	3 (1%)
49	Leksand	3 (1%)
50	Lessebo	4 (1%)

51	Lidingö	3 (1%)
52	Ludvika	3 (1%)
53	Luleå	2 (0%)
54	Lund	1 (0%)
55	Malmö	1 (0%)
56	Mora	1 (0%)
57	Mönsterås	4 (1%)
58	Mörbylånga	1 (0%)
59	Nordanstig	2 (0%)
60	Norrtälje	2 (0%)
61	Nybro	4 (1%)
62	Nykvarn	1 (0%)
63	Nyköping	1 (0%)
64	Nynäshamn	1 (0%)
65	Nässjö	3 (1%)
66	Olufström	4 (1%)
67	Osby	1 (0%)
68	Oskarshamn	1 (0%)
69	Oxelösund	2 (0%)
70	Pajala	1 (0%)
71	Piteå	4 (1%)
72	Ronneby	5 (1%)
73	Salem	6 (1%)
74	Sandviken	1 (0%)
75	Sigtuna	1 (0%)
76	Smedjebacken	6 (1%)
77	Solna	2 (0%)
78	Staffanstorp	1 (0%)
79	Stockholm	9 (2%)
80	Strängnäs	3 (1%)
81	Svalöv	2 (0%)
82	Säter	3 (1%)
[illegible]	[illegible]	[illegible]

83	Sävsjö	6 (1%)
84	Söderhamn	1 (0%)
85	Södertälje	1 (0%)
86	Sölvesborg	3 (1%)
87	Tierp	1 (0%)
88	Tomelilla	1 (0%)
89	Torsås	1 (0%)
90	Tranås	3 (1%)
91	Trelleborg	1 (0%)
92	Trosa	2 (0%)
93	Täby	4 (1%)
94	Upplands Väsby	1 (0%)
95	Uppsala	2 (0%)
96	Uppvidinge	2 (0%)
97	Vallentuna	7 (1%)
98	Vansbro	1 (0%)
99	Varberg	1 (0%)
100	Vetlanda	1 (0%)
101	Vimmerby	3 (1%)
102	Vingåker	3 (1%)
103	Värmdö	2 (0%)
104	Värnamo	2 (0%)
105	Västervik	2 (0%)
106	Växjö	2 (0%)
107	Ystad	2 (0%)
108	Älmhult	1 (0%)
109	Älvdalen	1 (0%)
110	Älvkarleby	1 (0%)
111	Östersund	3 (1%)
112	Österåker	2 (0%)
113	Östra Göinge	2 (0%)
114	Överkalix	4 (1%)
115	Ale	1 (0%)

116	Alingsås	2 (0%)
117	Arvika	2 (0%)
118	Askersund	5 (1%)
119	Bengtsfors	1 (0%)
120	Bjurholm	3 (1%)
121	Bollebygd	2 (0%)
122	Degerfors	1 (0%)
123	Dorotea	3 (1%)
124	Fagersta	3 (1%)
125	Falköping	1 (0%)
126	Forshaga	1 (0%)
127	Färgelanda	2 (0%)
128	Grums	1 (0%)
129	Grästorp	1 (0%)
130	Gullspång	1 (0%)
131	Göteborg	2 (0%)
132	Götene	5 (1%)
133	Hagfors	1 (0%)
134	Hallsberg	1 (0%)
135	Hallstahammar	3 (1%)
136	Hammarö	2 (0%)
137	Hjo	2 (0%)
138	Härnösand	1 (0%)
139	Härryda	2 (0%)
140	Karlsborg	1 (0%)
141	Karlskoga	3 (1%)
142	Karlstad	1 (0%)
143	Kinda	1 (0%)
144	Kramfors	1 (0%)
145	Kristinehamn	2 (0%)
146	Kungsör	1 (0%)
147	Kungälv	2 (0%)

148 Köping	2 (0%)
149 Laxå	1 (0%)
150 Lekeberg	2 (0%)
151 Lerum	2 (0%)
152 Lilla Edet	3 (1%)
153 Linköping	4 (1%)
154 Ljusnarsberg	1 (0%)
155 Lycksele	1 (0%)
156 Lysekil	3 (1%)
157 Mark	4 (1%)
158 Mjölby	3 (1%)
159 Motala	2 (0%)
160 Munkedal	1 (0%)
161 Munkfors	1 (0%)
162 Mölndal	3 (1%)
163 Norberg	1 (0%)
164 Nordmaling	1 (0%)
165 Norrköping	2 (0%)
166 Orust	5 (1%)
167 Robertsfors	1 (0%)
168 Sala	3 (1%)
169 Skara	1 (0%)
170 Skellefteå	4 (1%)
171 Skinnskatteberg	1 (0%)
172 Skövde	7 (1%)
173 Sollefteå	5 (1%)
174 Stenungsund	2 (0%)
175 Storuman	1 (0%)
176 Strömstad	2 (0%)
177 Sundsvall	3 (1%)
178 Sunne	2 (0%)
179 Surahammar	1 (0%)
180 Svenljunga	1 (0%)

[illegible]	Svenljunga	1 (0%)
181	Sötenäs	2 (0%)
182	Tanum	2 (0%)
183	Tidaholm	4 (1%)
184	Timrå	1 (0%)
185	Tjörn	1 (0%)
186	Tranemo	1 (0%)
187	Trollhättan	4 (1%)
188	Uddevalla	3 (1%)
189	Ulricehamn	5 (1%)
190	Umeå	5 (1%)
191	Valdemarsvik	2 (0%)
192	Vara	2 (0%)
193	Vilhemina	3 (1%)
194	Vindeln	1 (0%)
195	Vänersborg	4 (1%)
196	Vännäs	2 (0%)
197	Västerås	1 (0%)
198	Ydre	2 (0%)
199	Åmål	1 (0%)
200	Årjäng	3 (1%)
201	Öckerö	2 (0%)
202	Örebro	2 (0%)
203	Örnsköldsvik	1 (0%)
Svar		467

Län BGD

Sida 1 - Fråga 7 | Om kommunens/länets strategi för biologisk mångfald

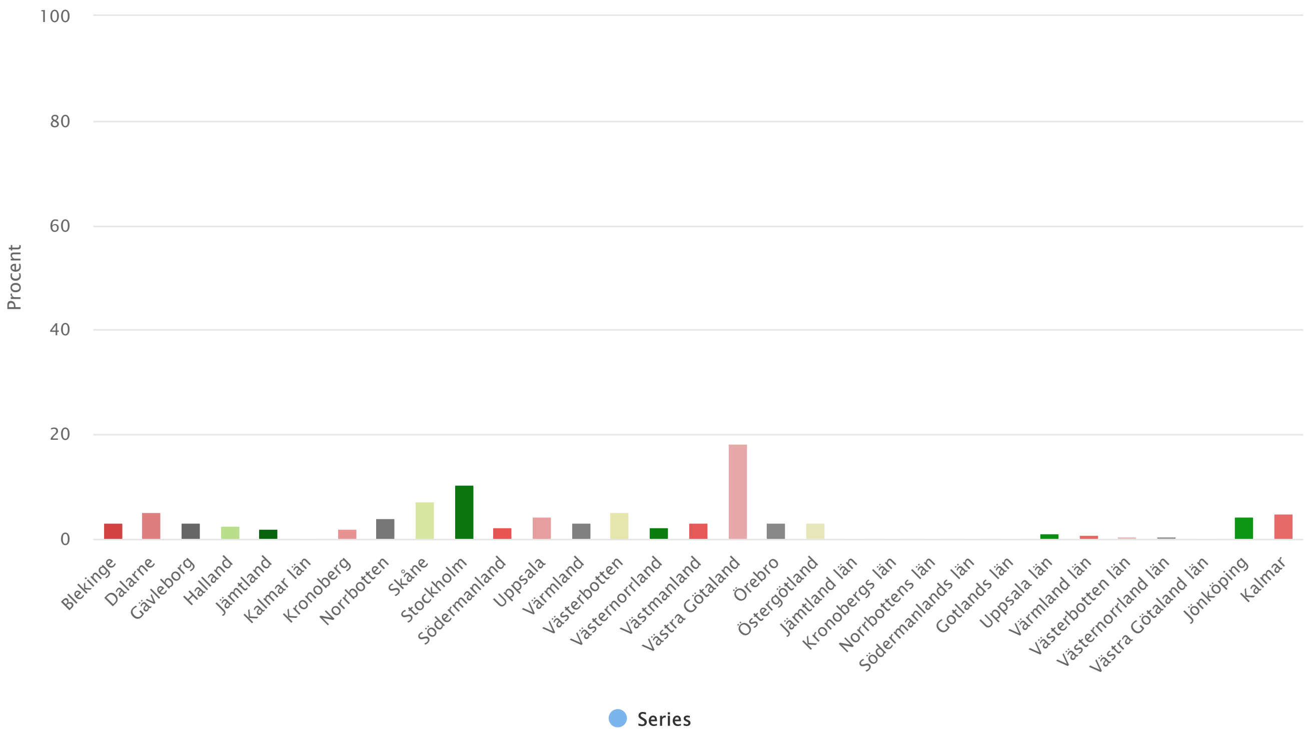

1 Blekinge	16 (3%)
2 Dalarne	25 (5%)
3 Gävleborg	16 (3%)
4 Halland	13 (3%)
5 Jämtland	10 (2%)
6 Kalmar län	1 (0%)
7 Kronoberg	10 (2%)
8 Norrbotten	20 (4%)
9 Skåne	36 (7%)
10 Stockholm	51 (10%)
11 Södermanland	12 (2%)
12 Uppsala	21 (4%)
13 Värmland	16 (3%)
14 Västerbotten	25 (5%)
15 Västernorrland	12 (2%)
16 Västmanland	16 (3%)
17 Västra Götaland	90 (18%)
18 Örebro	16 (3%)

[illegible]	[illegible]
19 Östergötland	16 (3%)
20 Jämtland län	1 (0%)
21 Kronobergs län	2 (0%)
22 Norrbottens län	1 (0%)
23 Södermanlands län	1 (0%)
24 Gotlands län	1 (0%)
25 Uppsala län	6 (1%)
26 Värmland län	4 (1%)
27 Västerbotten län	3 (1%)
28 Västernorrland län	3 (1%)
29 Västra Götaland län	1 (0%)
30 Jönköping	22 (4%)
31 Kalmar	24 (5%)
Svar	491

Kön (namn) BGD

Sida 1 - Fråga 8 | Om kommunens/länets strategi för biologisk mångfald

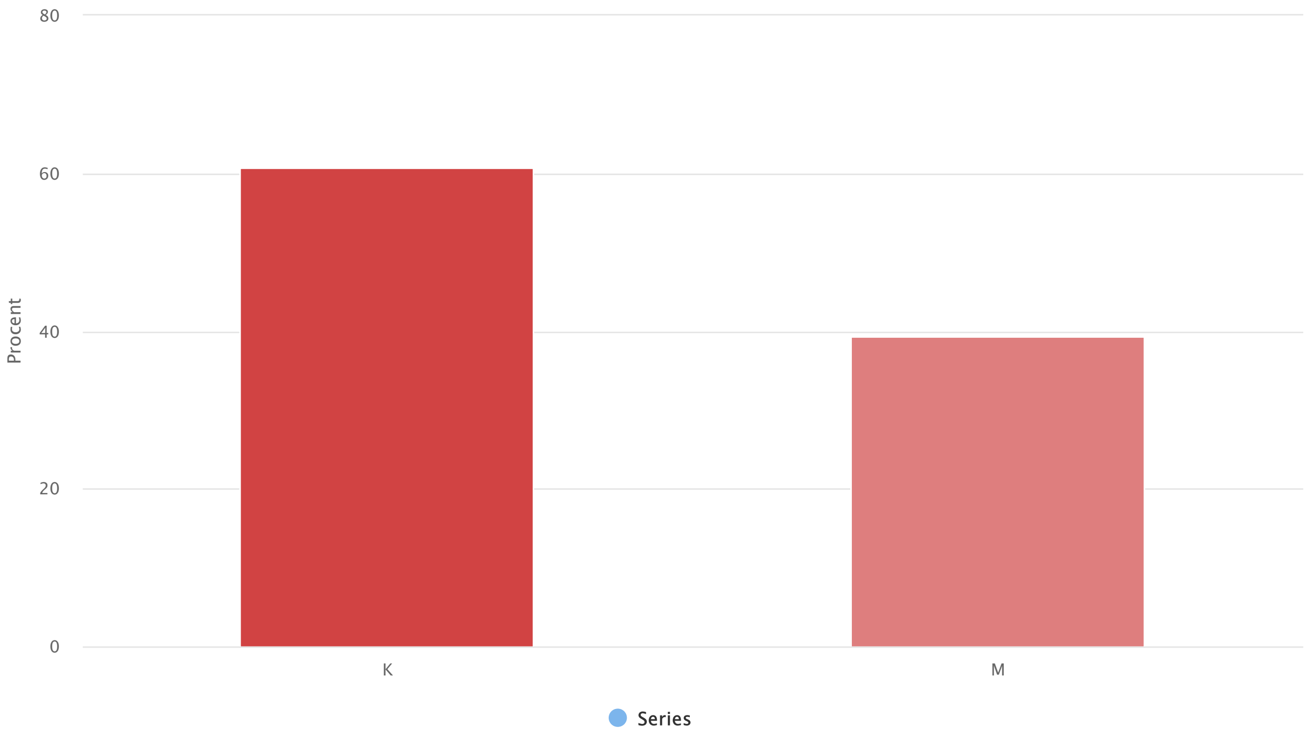

1 K	288 (61%)
2 M	187 (39%)
Svar	475

Är din kommun eller ditt län skogsägare?

Sida 1 - Fråga 9 | Om kommunens/länets strategi för biologisk mångfald

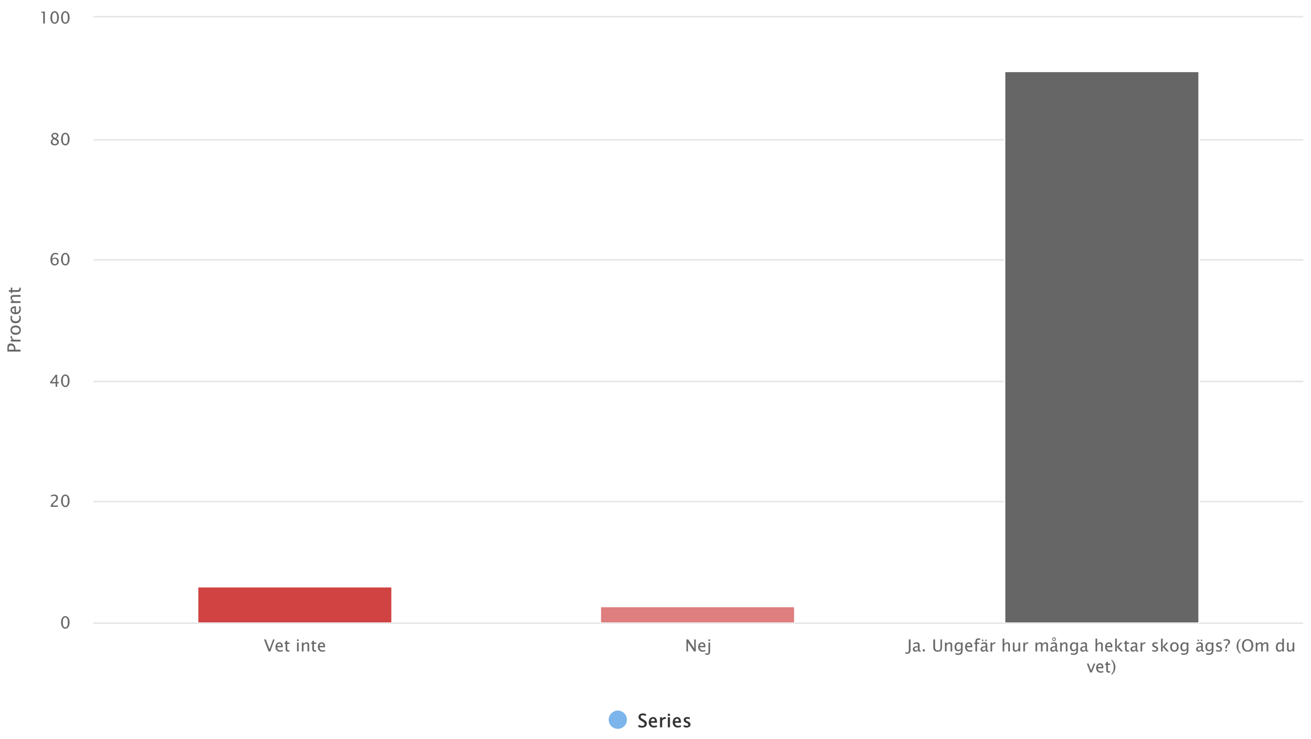

1 Vet inte	17 (6%)
2 Nej	8 (3%)
3 Ja. Ungefär hur många hektar skog ägs? (Om du vet)	259 (91%)
Svar	284

Ja. Ungefär hur många hektar skog ägs? (Om du vet)

Sida 1 - Fråga 10 | Om kommunens/länets strategi för biologisk mångfald

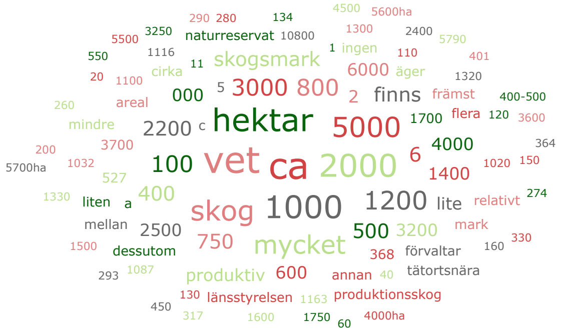

Vem sköter skogen?

Sida 1 - Fråga 11 | Om kommunens/länets strategi för biologisk mångfald

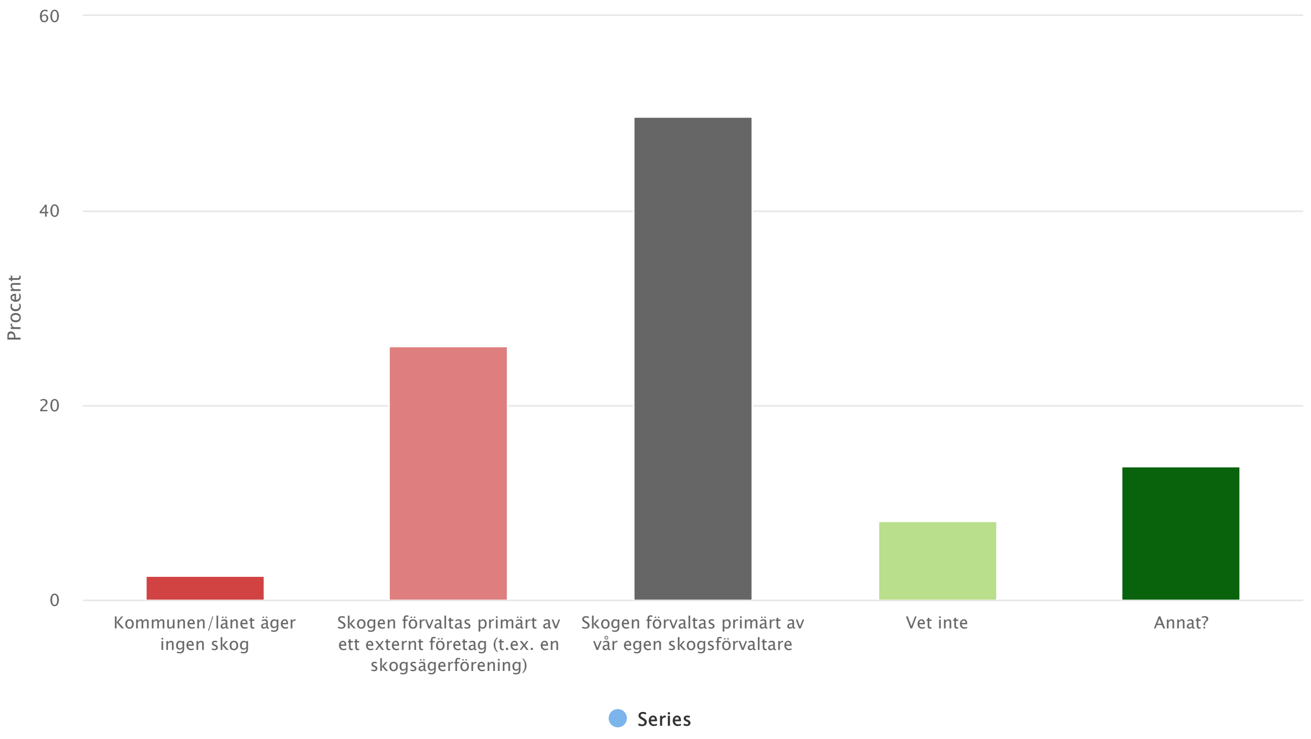

1 Kommunen/länet äger ingen skog	7 (2%)
2 Skogen förvaltas primärt av ett externt företag (t.ex. en skogsägerförening)	74 (26%)
3 Skogen förvaltas primärt av vår egen skogsförvaltare	141 (50%)
4 Vet inte	23 (8%)
5 Annat?	39 (14%)
Svar	284

Annat?

Sida 1 - Fråga 12 | Om kommunens/länets strategi för biologisk mångfald

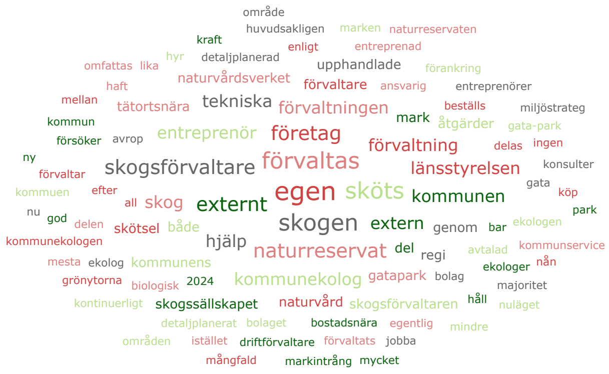

Vad är målet med skogsförvaltningen? Flera kryss kan sättas.

Sida 1 - Fråga 13 | Om kommunens/länets strategi för biologisk mångfald

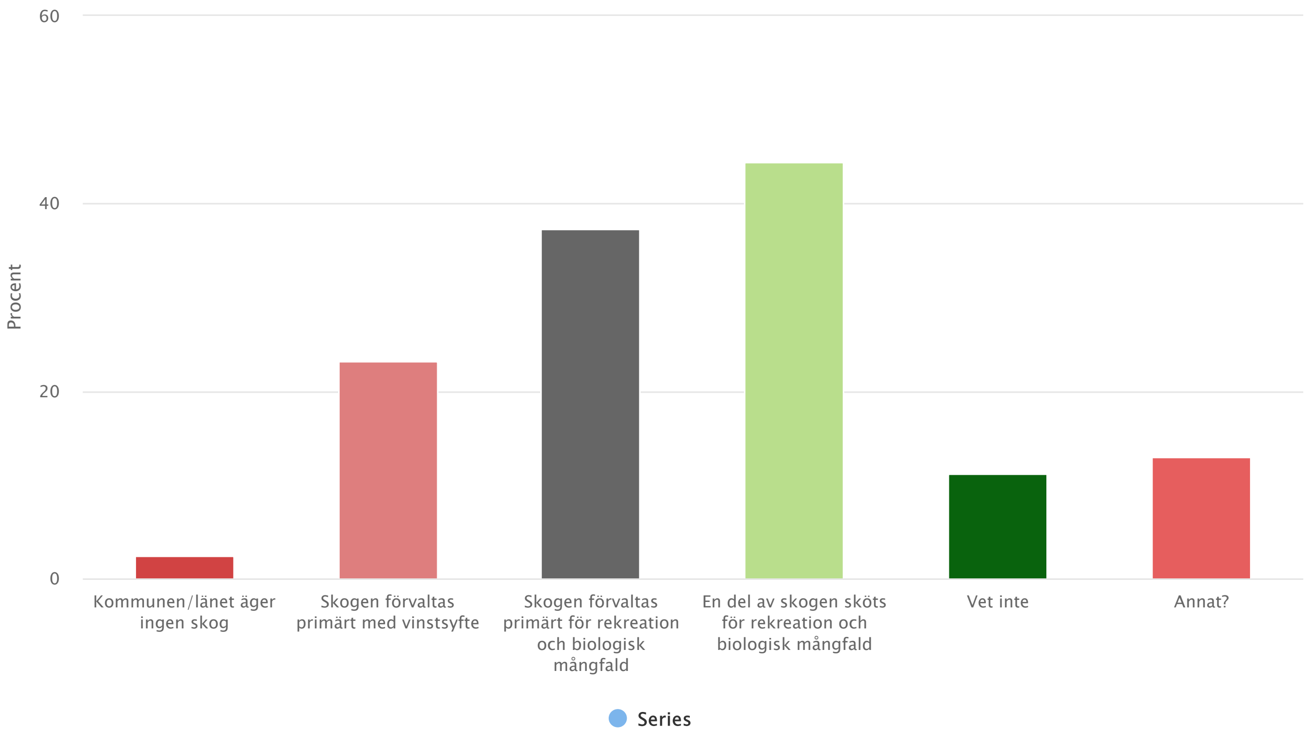

1 Kommunen/länet äger ingen skog	7 (2%)
2 Skogen förvaltas primärt med vinstsyfte	66 (23%)
3 Skogen förvaltas primärt för rekreation och biologisk mångfald	106 (37%)
4 En del av skogen sköts för rekreation och biologisk mångfald	126 (44%)
5 Vet inte	32 (11%)
6 Annat?	37 (13%)
Svar	284

Annat?

Sida 1 - Fråga 14 | Om kommunens/länets strategi för biologisk mångfald

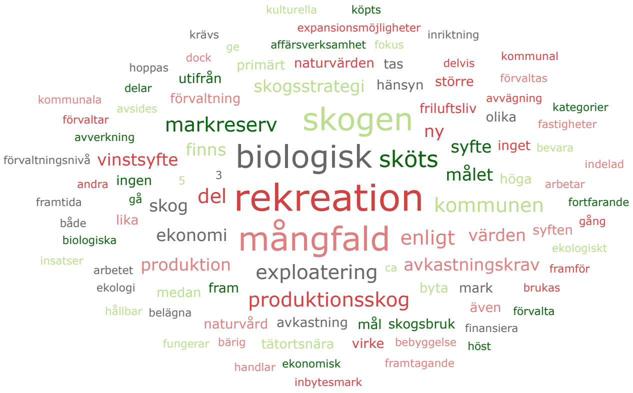

Strategiska insatser för biologisk mångfald på landskapsnivå

Sida 2 - Fråga 1 | Strategiska insatser för biologisk mångfald på landskapsnivå

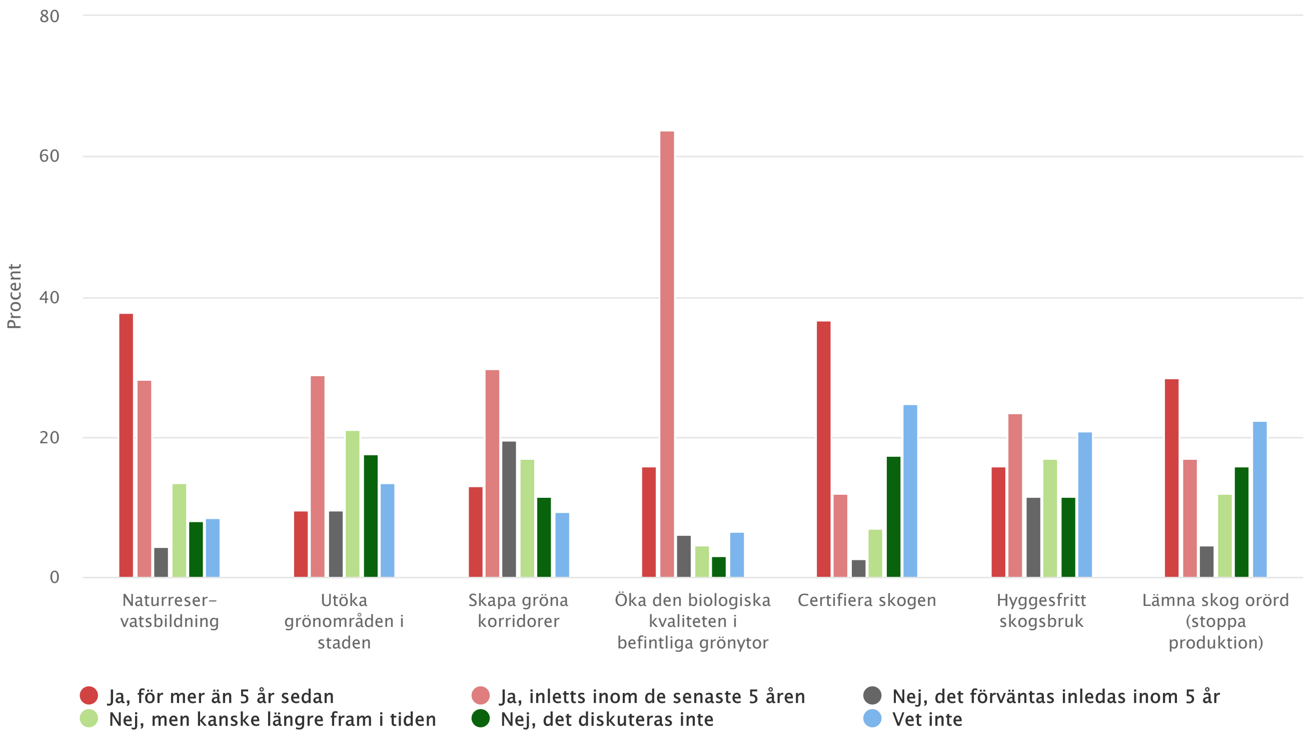

	Ja, för mer än 5 år sedan	Ja, inletts inom de senaste 5 åren	Nej, det förväntas inledas inom 5 år	Nej, men kanske längre fram i tiden	Nej, det diskuteras inte
Naturreser-vatsbildning	**98 (38%)**	73 (28%)	11 (4%)	35 (13%)	21 (8%)
Utöka grönområden i staden	25 (10%)	**75 (29%)**	25 (10%)	55 (21%)	46 (18%)
Skapa gröna korridorer	34 (13%)	**77 (30%)**	51 (20%)	44 (17%)	30 (12%)
Öka den biologiska kvaliteten i befintliga grönytor	41 (16%)	**166 (64%)**	16 (6%)	12 (5%)	8 (3%)
Certifiera skogen	**95 (37%)**	31 (12%)	7 (3%)	18 (7%)	45 (17%)
Hyggesfritt skogsbruk	41 (16%)	**61 (23%)**	30 (12%)	44 (17%)	30 (12%)
Lämna skog orörd (stoppa produktion)	**74 (28%)**	44 (17%)	12 (5%)	31 (12%)	41 (16%)

	Vet inte	Svar
Naturreser-vatsbildning	22 (8%)	**260**
Utöka grönområden i staden	35 (13%)	**261**
Skapa gröna korridorer	24 (9%)	**260**
Öka den biologiska kvaliteten i befintliga grönytor	17 (7%)	**260**
Certifiera skogen	64 (25%)	**260**
Hyggesfritt skogsbruk	54 (21%)	**260**
Lämna skog orörd (stoppa produktion)	58 (22%)	**260**

Andra insatser på landskapsnivå?

Sida 2 - Fråga 2 | Strategiska insatser för biologisk mångfald på landskapsnivå

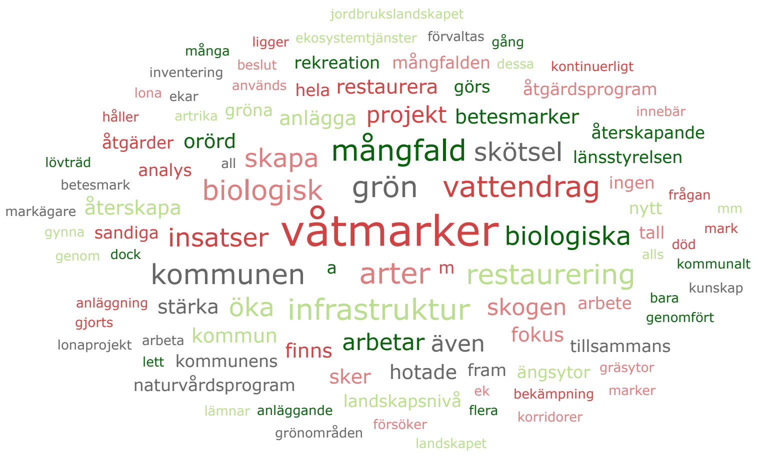

Strategiska åtgärder för att främja biologisk mångfald på platsen

Sida 3 - Fråga 1 | Strategiska åtgärder för att främja biologisk mångfald på platsen

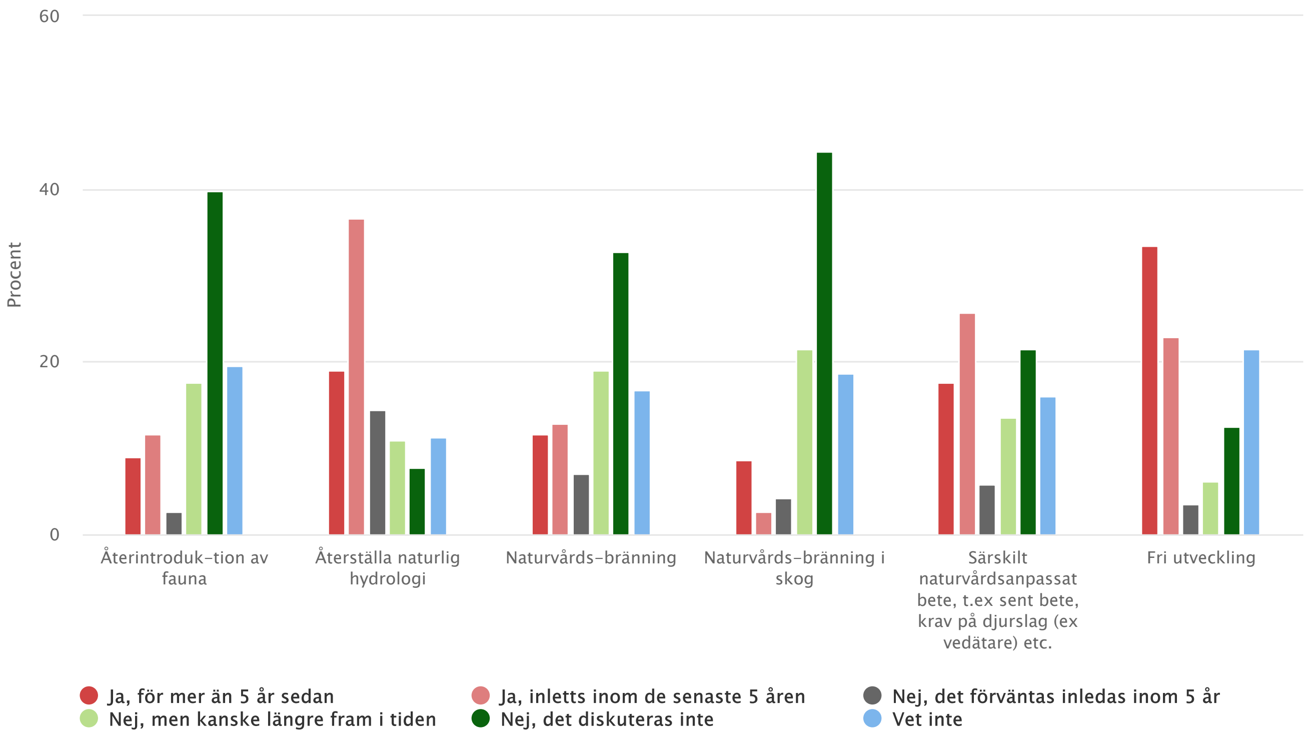

	Ja, för mer än 5 år sedan	Ja, inletts inom de senaste 5 åren	Nej, det förväntas inledas inom 5 år	Nej, men kanske längre fram i tiden	Nej, det diskuteras inte
Återintroduk-tion av fauna	23 (9%)	30 (12%)	7 (3%)	45 (18%)	**102 (40%)**
Återställa naturlig hydrologi	49 (19%)	**94 (37%)**	37 (14%)	28 (11%)	20 (8%)
Naturvårds-bränning	30 (12%)	33 (13%)	18 (7%)	49 (19%)	**84 (33%)**
Naturvårds-bränning i skog	22 (9%)	7 (3%)	11 (4%)	55 (21%)	**114 (44%)**
Särskilt naturvårdsanpassat bete, t.ex sent bete, krav på djurslag (ex vedätare) etc.	45 (18%)	**66 (26%)**	15 (6%)	35 (14%)	55 (21%)
Fri utveckling	**86 (33%)**	59 (23%)	9 (4%)	16 (6%)	32 (12%)

	Vet inte	Svar
Återintroduk-tion av fauna	50 (19%)	**257**
Återställa naturlig hydrologi	29 (11%)	**257**
Naturvårds-bränning	43 (17%)	**257**
Naturvårds-bränning i skog	48 (19%)	**257**
Särskilt naturvårdsanpassat bete, t.ex sent bete, krav på djurslag (ex vedätare) etc.	41 (16%)	**257**
Fri utveckling	55 (21%)	**257**

Andra åtgärder för att initiera naturliga processer?

Sida 3 - Fråga 2 | Strategiska åtgärder för att främja biologisk mångfald på platsen

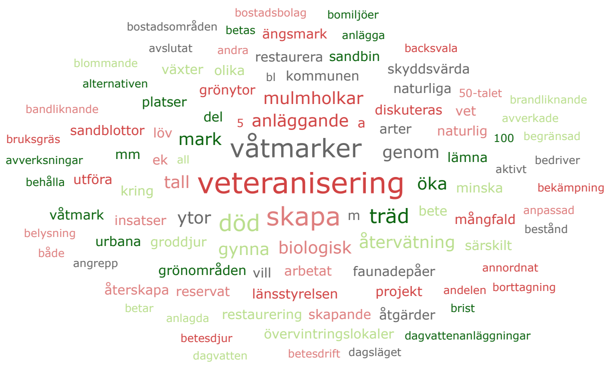

1. Vårda/Bevara befintliga naturtyper och element: *Hed, myr, rikkärr, ljusöppen skog/gräsmark, våtmark, evighetsträd etc.*
2. Skapa mikrohabitat: *Mulmholk, faunadepå, veteranisering av träd, stenhög, groddjursdamm, markstörning, lämna död ved etc.*
3. Skapa mer växtmångfald: *Omställning av klippta gräsytor till ängsmark/högväxta gräsytor, användning av inhemska växtarter, insamling av lokala frön, blomrika vägkanter, bekämpa invasiva arter, etablera skogsbryn, sommarbete eller slåtter på ängar, etc.*
4. Initiera naturliga processer: *Återintroduktion av fauna eller skogsbrand, återställa naturlig hydrologi, naturvårdsanpassat bete, användning av naturlig tillväxt, etc.*
5. Inventering av biologisk mångfald
6. Förmedling av arbetet för biologisk mångfald till medborgare och institutioner: *Hemsida, skyltning, föreläsningar, naturguidning etc.*

Sida 4 - Fråga 1 | Operationella åtgärder

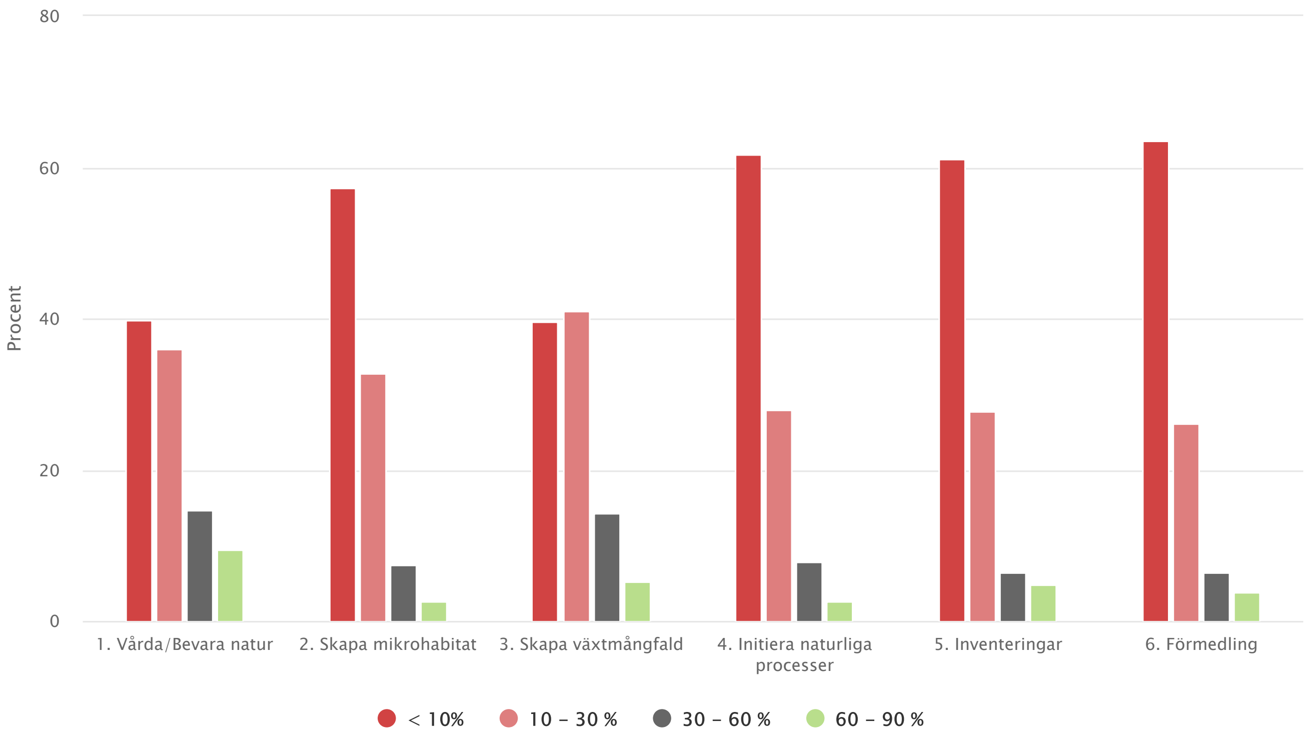

	< 10%	10 - 30 %	30 - 60 %	60 - 90 %
1. Vårda/Bevara natur	**92 (40%)**	83 (36%)	34 (15%)	22 (10%)
2. Skapa mikrohabitat	**131 (57%)**	75 (33%)	17 (7%)	6 (3%)
3. Skapa växtmångfald	92 (40%)	**95 (41%)**	33 (14%)	12 (5%)
4. Initiera naturliga processer	**143 (62%)**	65 (28%)	18 (8%)	6 (3%)
5. Inventeringar	**141 (61%)**	64 (28%)	15 (6%)	11 (5%)
6. Förmedling	**146 (63%)**	60 (26%)	15 (7%)	9 (4%)

	Svar
1. Vårda/Bevara natur	**231**
2. Skapa mikrohabitat	**229**
3. Skapa växtmångfald	**232**
4. Initiera naturliga processer	**232**
5. Inventeringar	**231**
6. Förmedling	**230**

Annat som använder betydande resurser?

Sida 4 - Fråga 2 | Operationella åtgärder

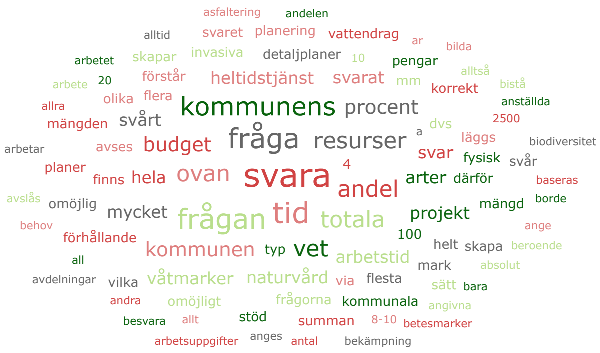

1. Markägare. Flera kryss kan sättas.

Sida 5 - Fråga 1 | Intressenter, aktörer och styrning

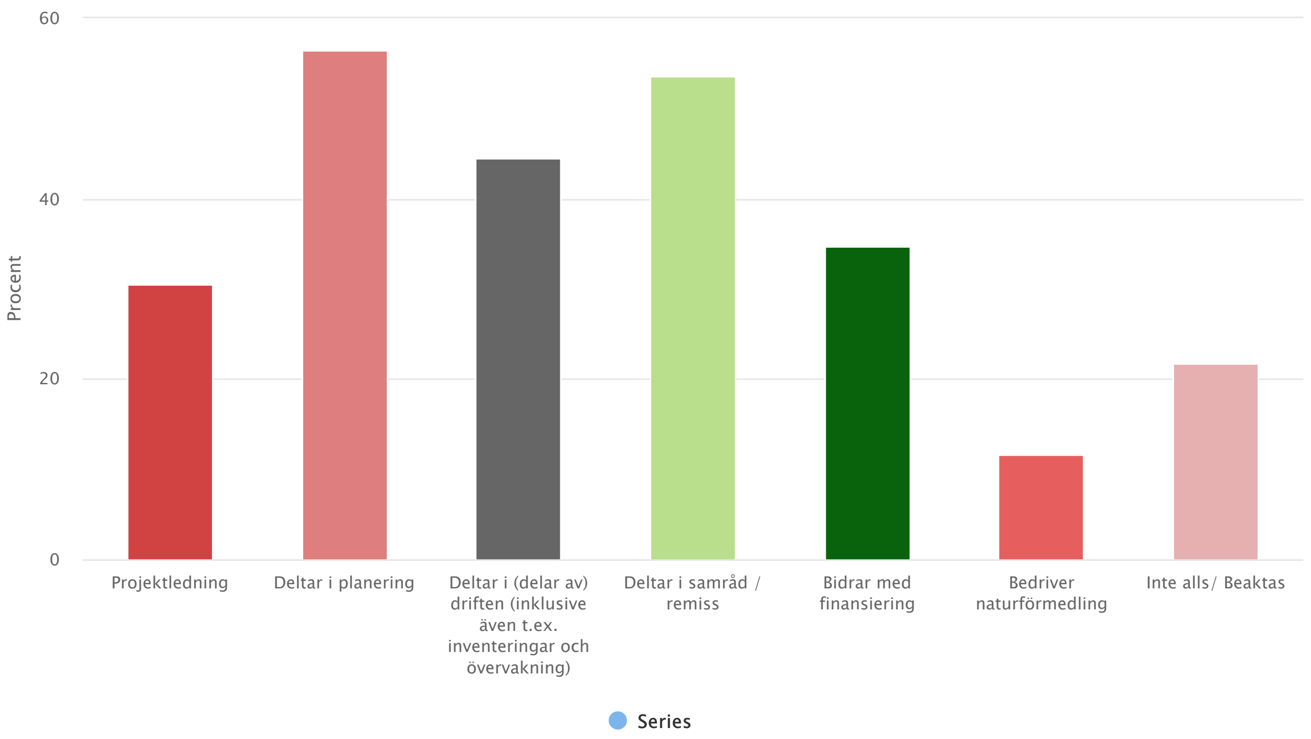

1 Projektledning	63 (30%)
2 Deltar i planering	117 (57%)
3 Deltar i (delar av) driften (inklusive även t.ex. inventeringar och övervakning)	92 (44%)
4 Deltar i samråd / remiss	111 (54%)
5 Bidrar med finansiering	72 (35%)
6 Bedriver naturförmedling	24 (12%)
7 Inte alls/ Beaktas	45 (22%)
Svar	207

2. Privata företag. Flera kryss kan sättas.

Sida 6 - Fråga 1 | Intressenter, aktörer och styrning

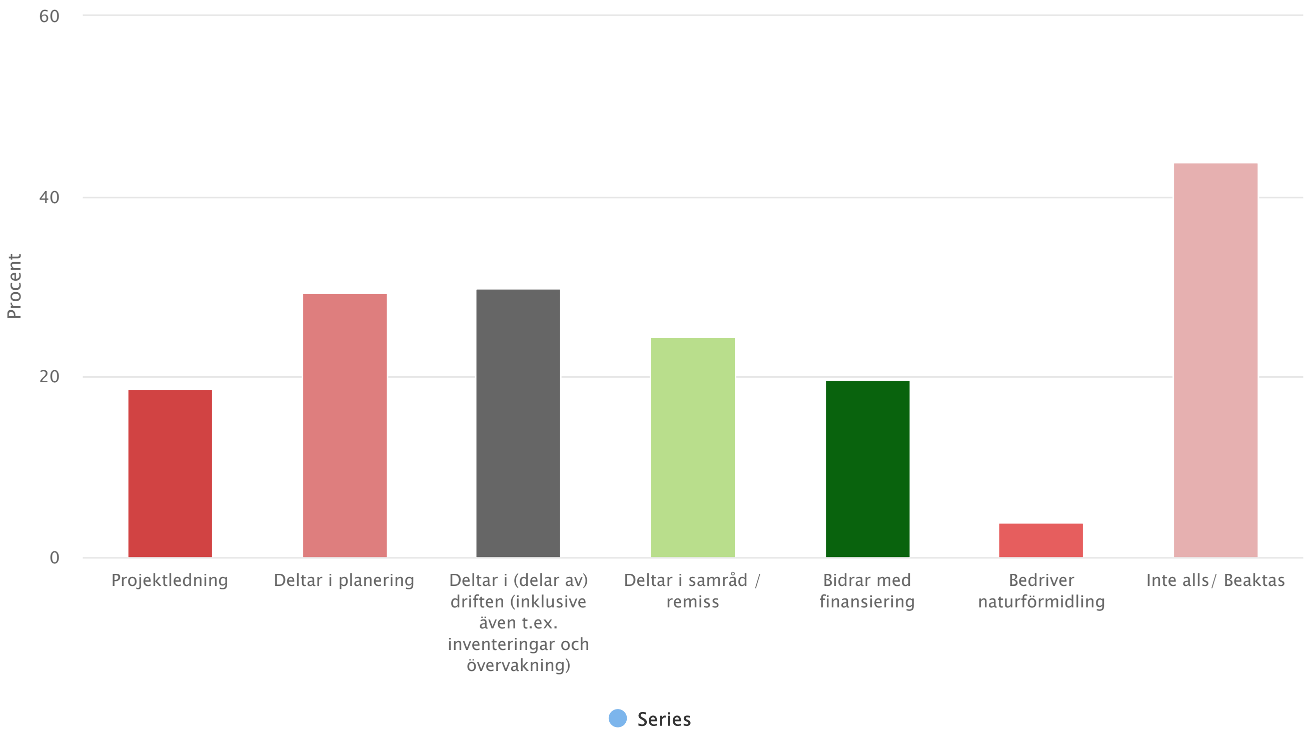

1 Projektledning	39 (19%)
2 Deltar i planering	61 (29%)
3 Deltar i (delar av) driften (inklusive även t.ex. inventeringar och övervakning)	62 (30%)
4 Deltar i samråd / remiss	51 (25%)
5 Bidrar med finansiering	41 (20%)
6 Bedriver naturförmidling	8 (4%)
7 Inte alls/ Beaktas	91 (44%)
Svar	208

3. Privata stiftelser. Flera kryss kan sättas.

Sida 7 - Fråga 1 | Intressenter, aktörer och styrning

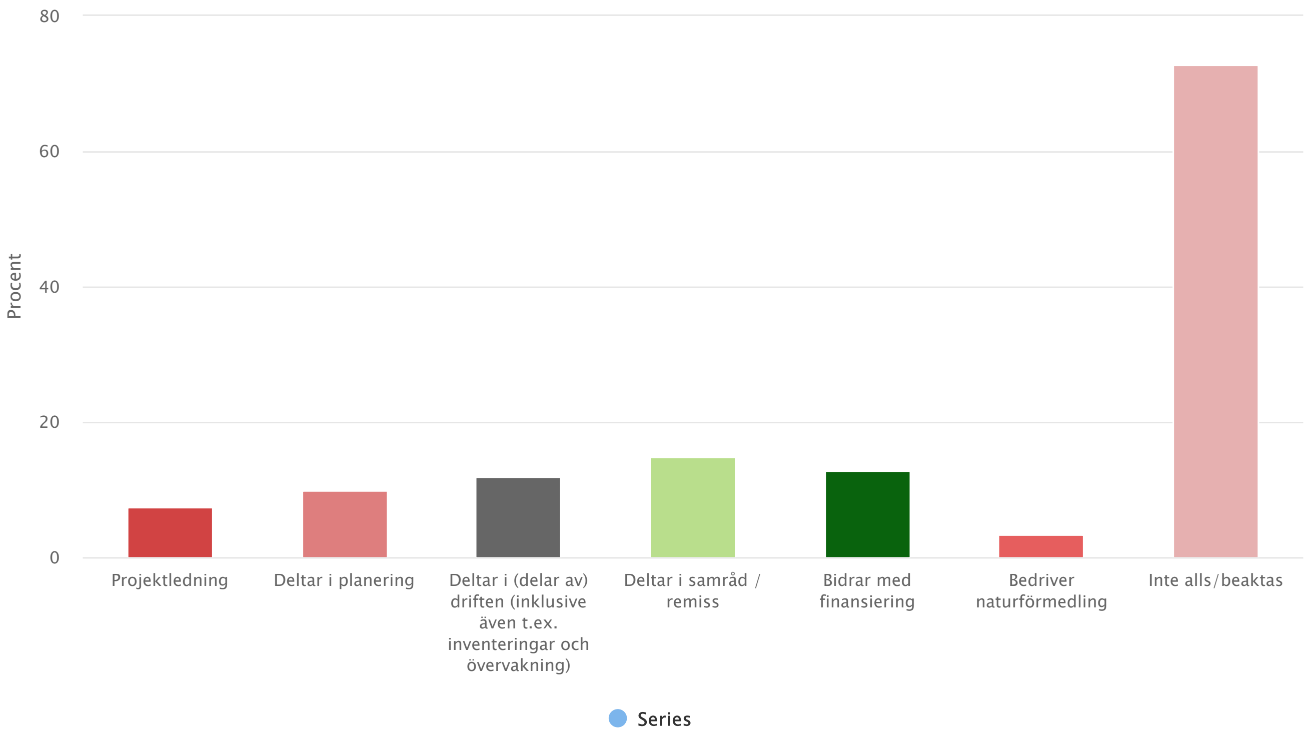

1 Projektledning	15 (7%)
2 Deltar i planering	20 (10%)
3 Deltar i (delar av) driften (inklusive även t.ex. inventeringar och övervakning)	24 (12%)
4 Deltar i samråd / remiss	30 (15%)
5 Bidrar med finansiering	26 (13%)
6 Bedriver naturförmedling	7 (3%)
7 Inte alls/beaktas	148 (73%)
Svar	203

4. Råd/styrgrupp av intressenter. Flera kryss kan sättas.

Sida 8 - Fråga 1 | Intressenter, aktörer och styrning

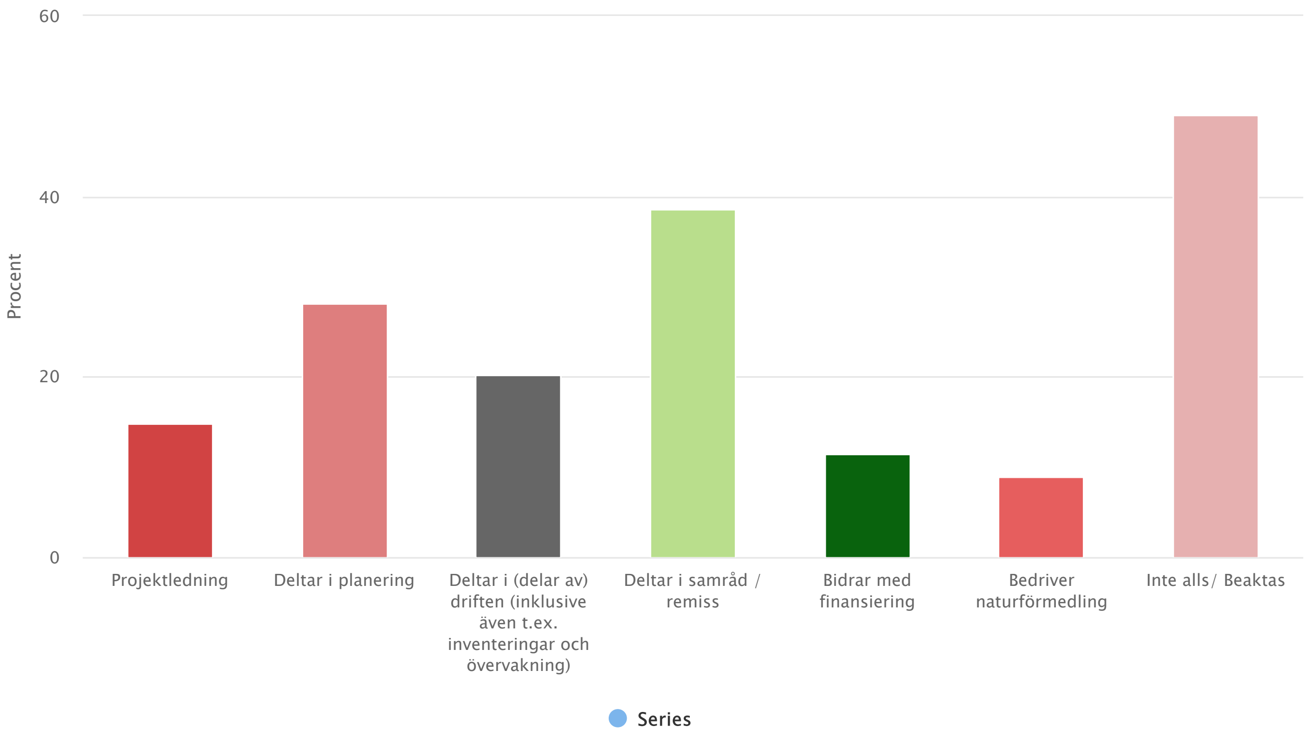

1 Projektledning	30 (15%)
2 Deltar i planering	57 (28%)
3 Deltar i (delar av) driften (inklusive även t.ex. inventeringar och övervakning)	41 (20%)
4 Deltar i samråd / remiss	78 (39%)
5 Bidrar med finansiering	23 (11%)
6 Bedriver naturförmedling	18 (9%)
7 Inte alls/ Beaktas	99 (49%)
Svar	202

5. Organiserade intressegrupper (t.ex. ornitologer, jägare, natur/miljö-organisationer, hembygdsföreningar, LRF). Flera kryss kan sättas.

Sida 9 - Fråga 1 | Intressenter, aktörer och styrning

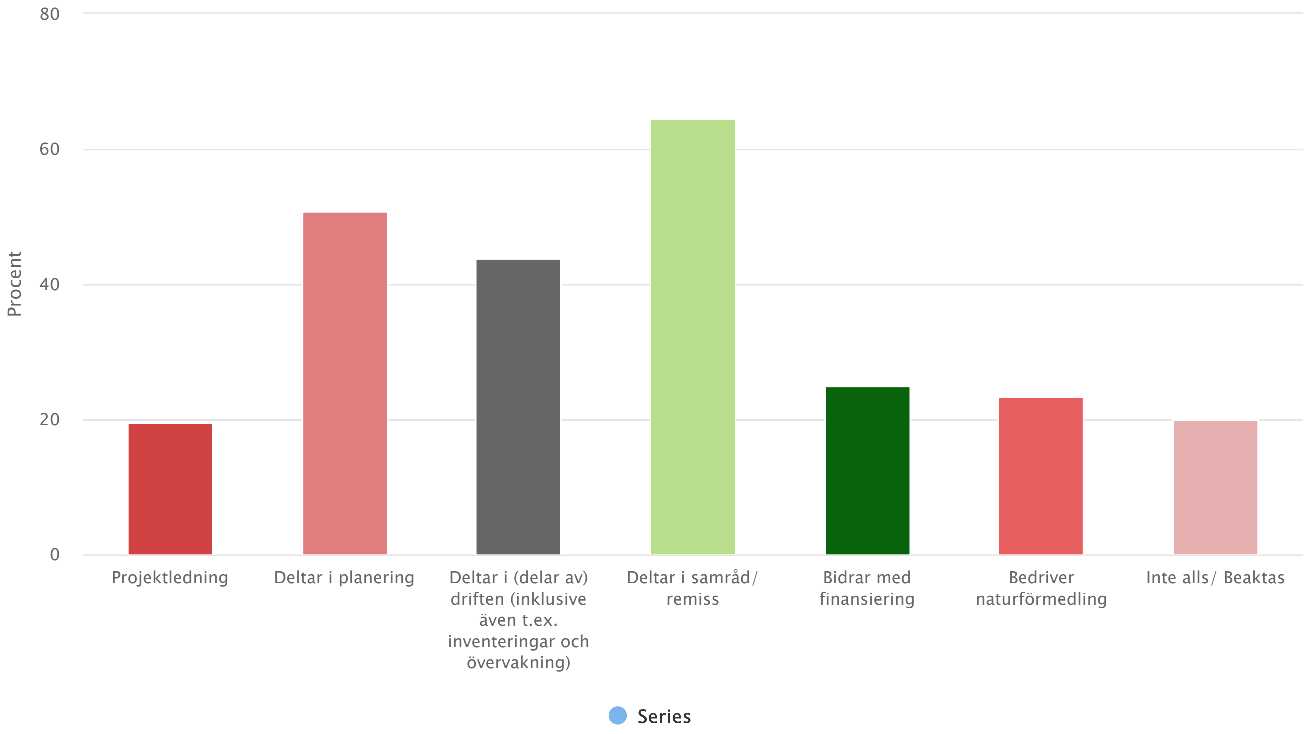

1 Projektledning	40 (20%)
2 Deltar i planering	104 (51%)
3 Deltar i (delar av) driften (inklusive även t.ex. inventeringar och övervakning)	90 (44%)
4 Deltar i samråd/ remiss	132 (64%)
5 Bidrar med finansiering	51 (25%)
6 Bedriver naturförmedling	48 (23%)
7 Inte alls/ Beaktas	41 (20%)
Svar	205

6. Skolor och andra lokala användargrupper (t.ex. barninstitutioner, idrottsföreningar, scouter, ryttare, mountainbikar etc.). Flera kryss kan sättas.

Sida 10 - Fråga 1 | Intressenter, aktörer och styrning

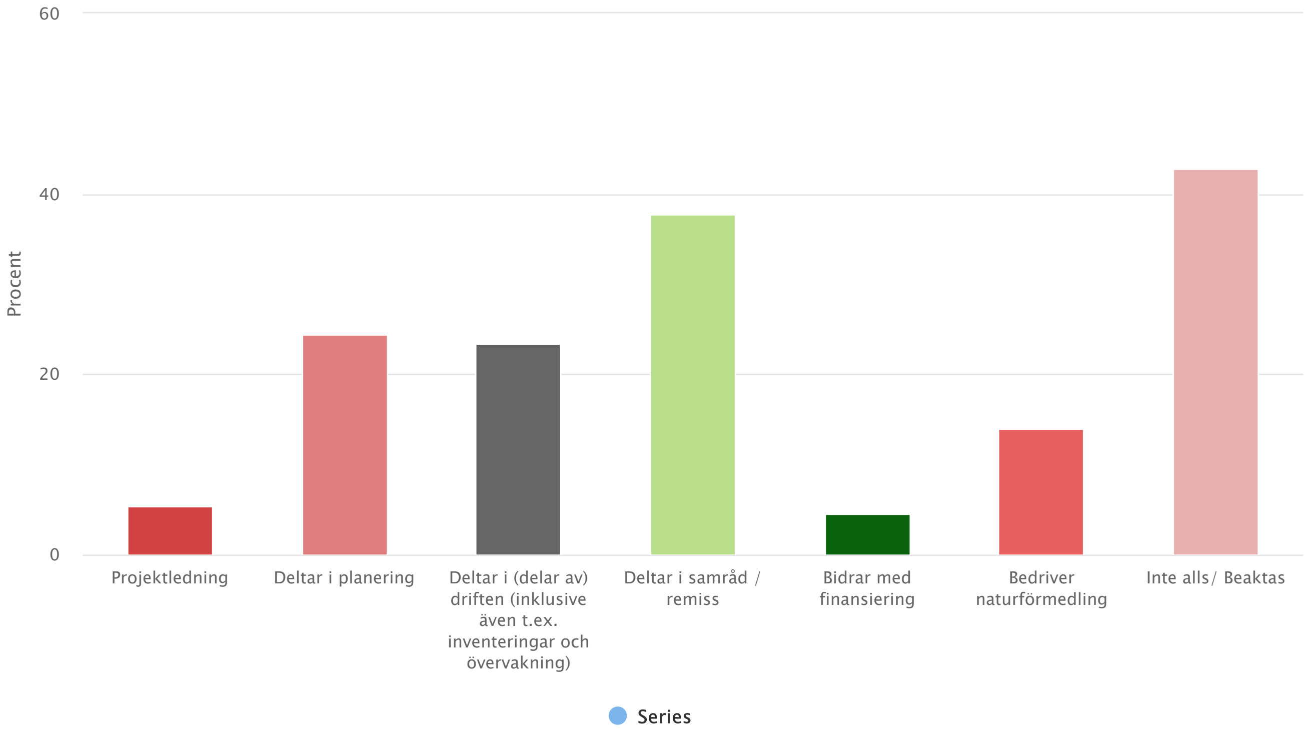

1 Projektledning	11 (5%)
2 Deltar i planering	49 (24%)
3 Deltar i (delar av) driften (inklusive även t.ex. inventeringar och övervakning)	47 (23%)
4 Deltar i samråd / remiss	76 (38%)
5 Bidrar med finansiering	9 (4%)
6 Bedriver naturförmedling	28 (14%)
7 Inte alls/ Beaktas	86 (43%)
Svar	201

7. Bostadsföreningar, Fastighetsbolag. Flera kryss kan sättas.

Sida 11 - Fråga 1 | Intressenter, aktörer och styrning

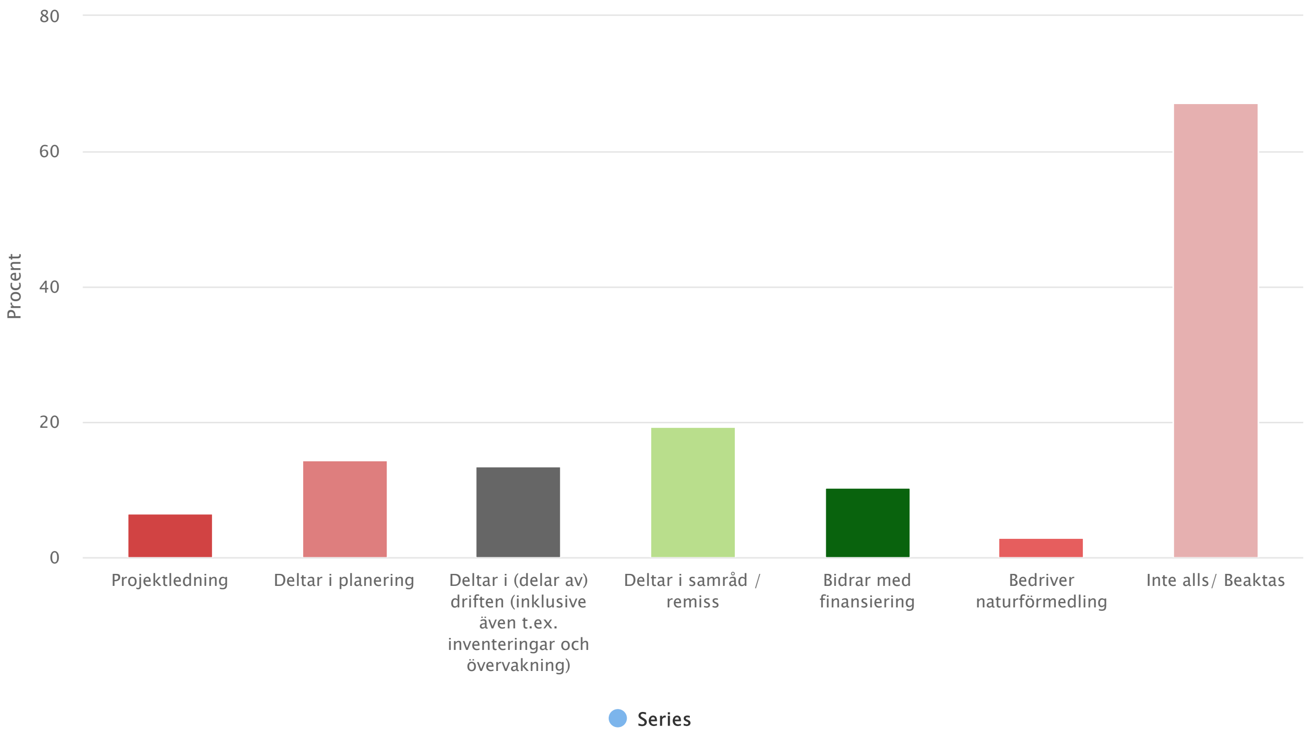

1 Projektledning	13 (6%)
2 Deltar i planering	29 (14%)
3 Deltar i (delar av) driften (inklusive även t.ex. inventeringar och övervakning)	27 (13%)
4 Deltar i samråd / remiss	39 (19%)
5 Bidrar med finansiering	21 (10%)
6 Bedriver naturförmedling	6 (3%)
7 Inte alls/ Beaktas	135 (67%)
Svar	201

8. Universitet/Högskolor. Flera kryss kan sättas.

Sida 12 - Fråga 1 | Intressenter, aktörer och styrning

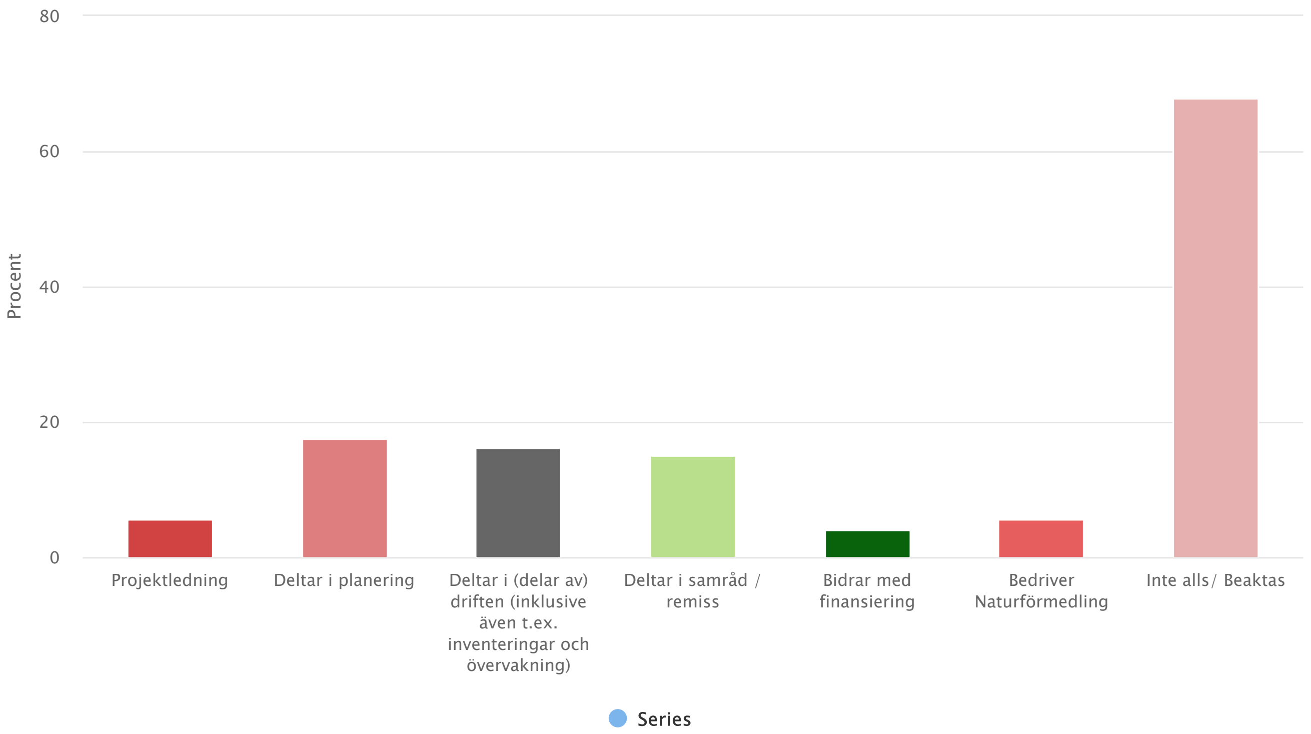

1 Projektledning	11 (6%)
2 Deltar i planering	35 (18%)
3 Deltar i (delar av) driften (inklusive även t.ex. inventeringar och övervakning)	32 (16%)
4 Deltar i samråd / remiss	30 (15%)
5 Bidrar med finansiering	8 (4%)
6 Bedriver Naturförmedling	11 (6%)
7 Inte alls/ Beaktas	135 (68%)
Svar	199

9. Privata rådgivare. Flera kryss kan sättas.

Sida 13 - Fråga 1 | Intressenter, aktörer och styrning

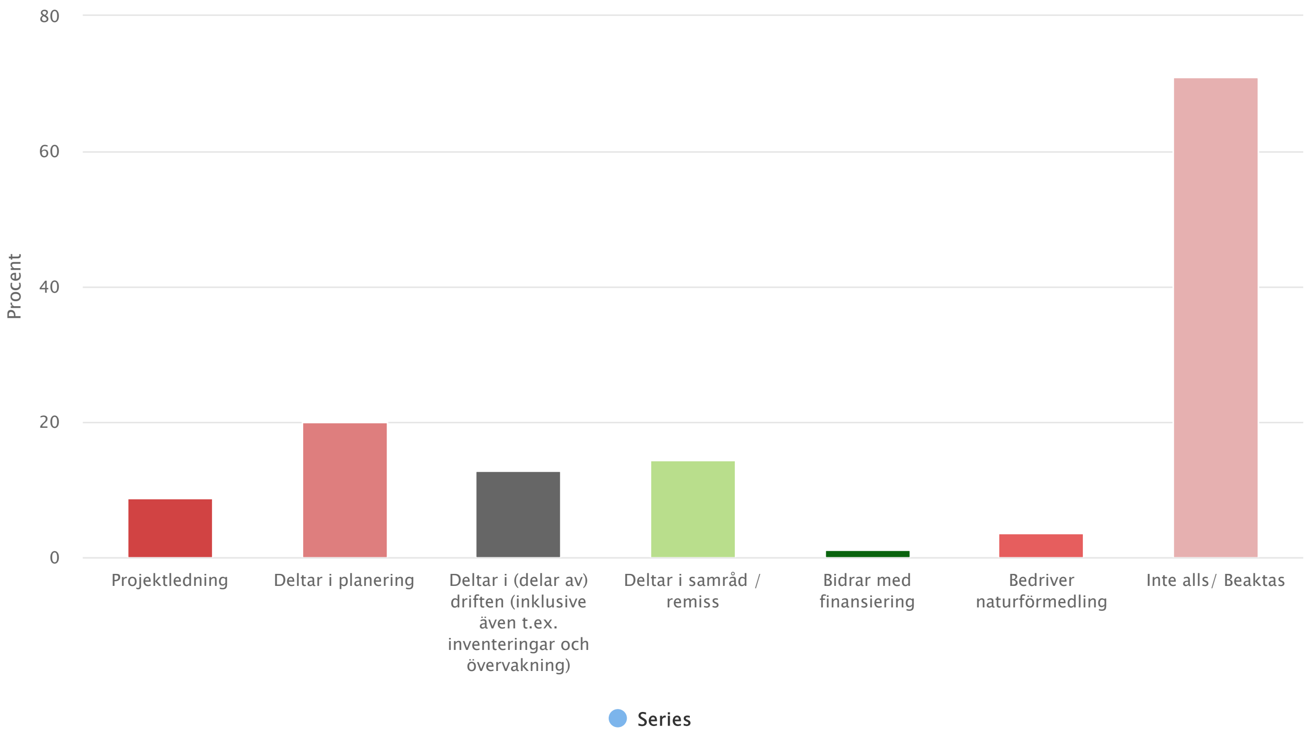

1 Projektledning	17 (9%)
2 Deltar i planering	39 (20%)
3 Deltar i (delar av) driften (inklusive även t.ex. inventeringar och övervakning)	25 (13%)
4 Deltar i samråd / remiss	28 (14%)
5 Bidrar med finansiering	2 (1%)
6 Bedriver naturförmedling	7 (4%)
7 Inte alls/ Beaktas	139 (71%)
Svar	196

10. Privata entreprenörer. Flera kryss kan sättas.

Sida 14 - Fråga 1 | Intressenter, aktörer och styrning

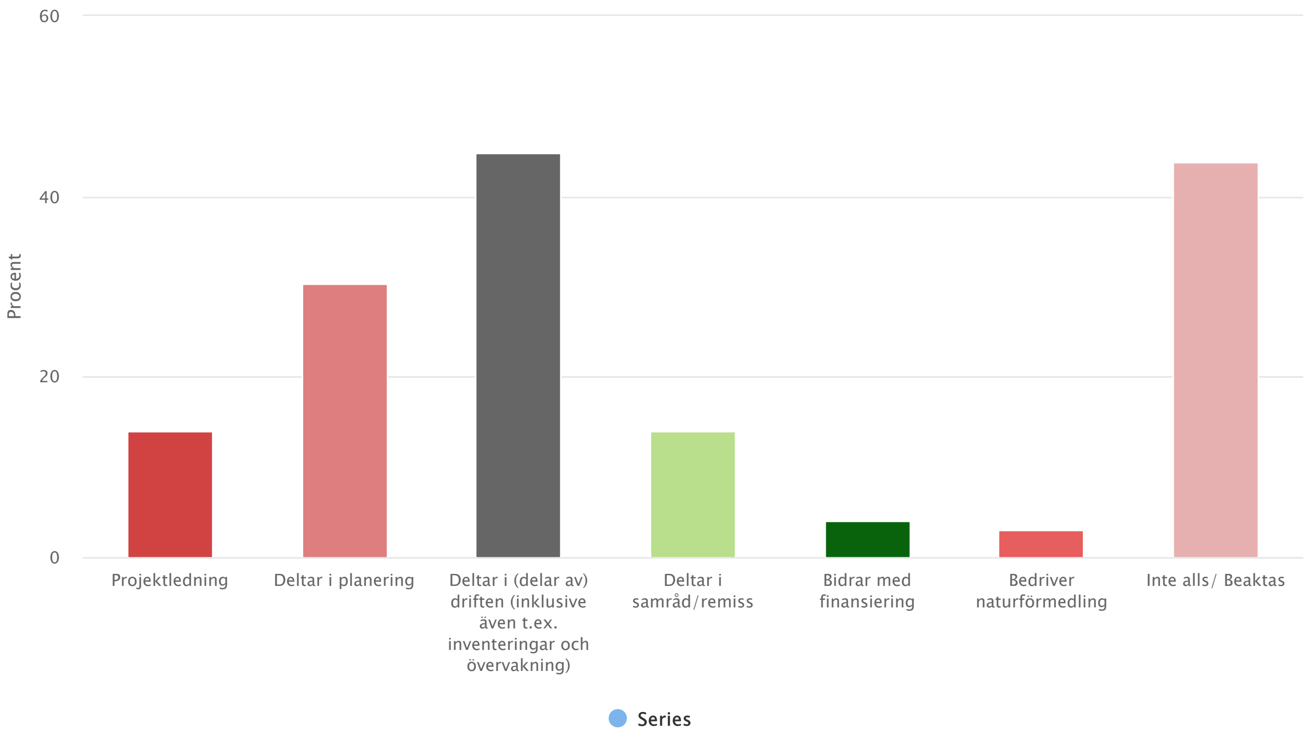

1 Projektledning	28 (14%)
2 Deltar i planering	61 (30%)
3 Deltar i (delar av) driften (inklusive även t.ex. inventeringar och övervakning)	90 (45%)
4 Deltar i samråd/remiss	28 (14%)
5 Bidrar med finansiering	8 (4%)
6 Bedriver naturförmedling	6 (3%)
7 Inte alls/ Beaktas	88 (44%)
Svar	201

Andra grupper? Hur är de involverade?

Sida 14 - Fråga 2 | Intressenter, aktörer och styrning

Vad är din roll inom din organisation? Välj den som passar bäst.

Sida 15 - Fråga 1 | Du som förvaltare av natur och biologisk mångfald

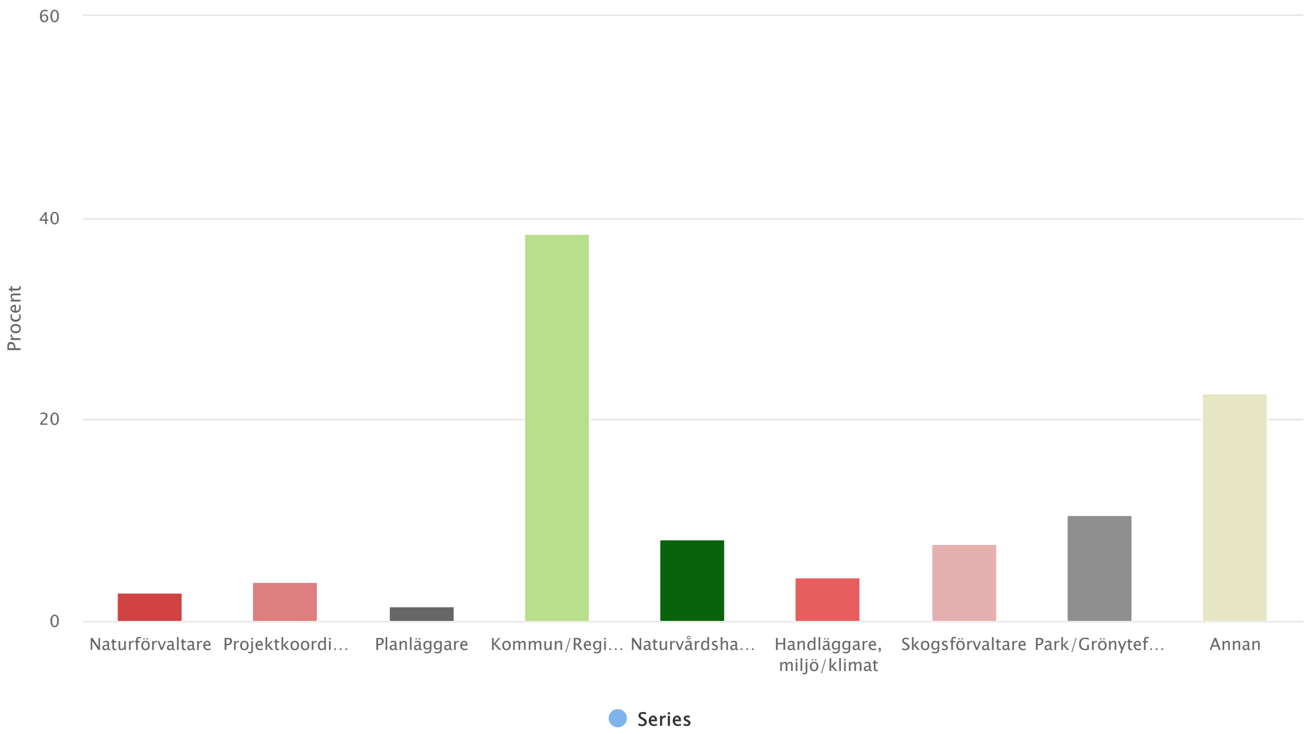

1 Naturförvaltare	6 (3%)
2 Projektkoordinator	8 (4%)
3 Planläggare	3 (1%)
4 Kommun/Regions-ekolog	80 (38%)
5 Naturvårdshandläggare	17 (8%)
6 Handläggare, miljö/klimat	9 (4%)
7 Skogsförvaltare	16 (8%)
8 Park/Grönyteförvaltare	22 (11%)
9 Annan	47 (23%)
Svar	208

Annan

Sida 15 - Fråga 2 | Du som förvaltare av natur och biologisk mångfald

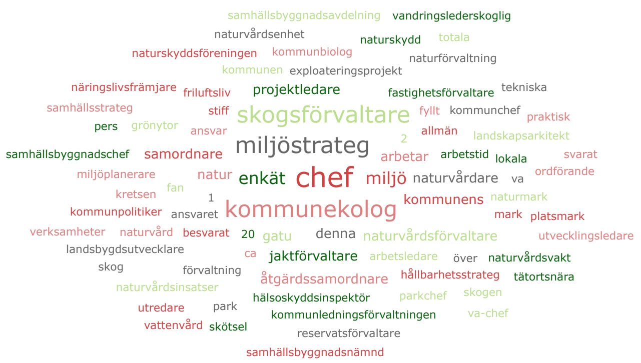

Hur länge har du varit i din nuvarande position? Skriv antal år

Sida 15 - Fråga 3 | Du som förvaltare av natur och biologisk mångfald

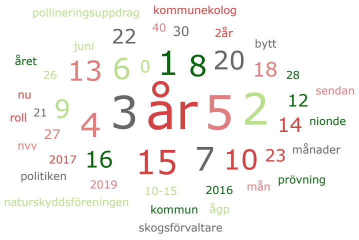

Vad har du för utbildningsbakgrund? Om du har flera, välj den du använder mest i ditt nuvarande arbete

Sida 15 - Fråga 4 | Du som förvaltare av natur och biologisk mångfald

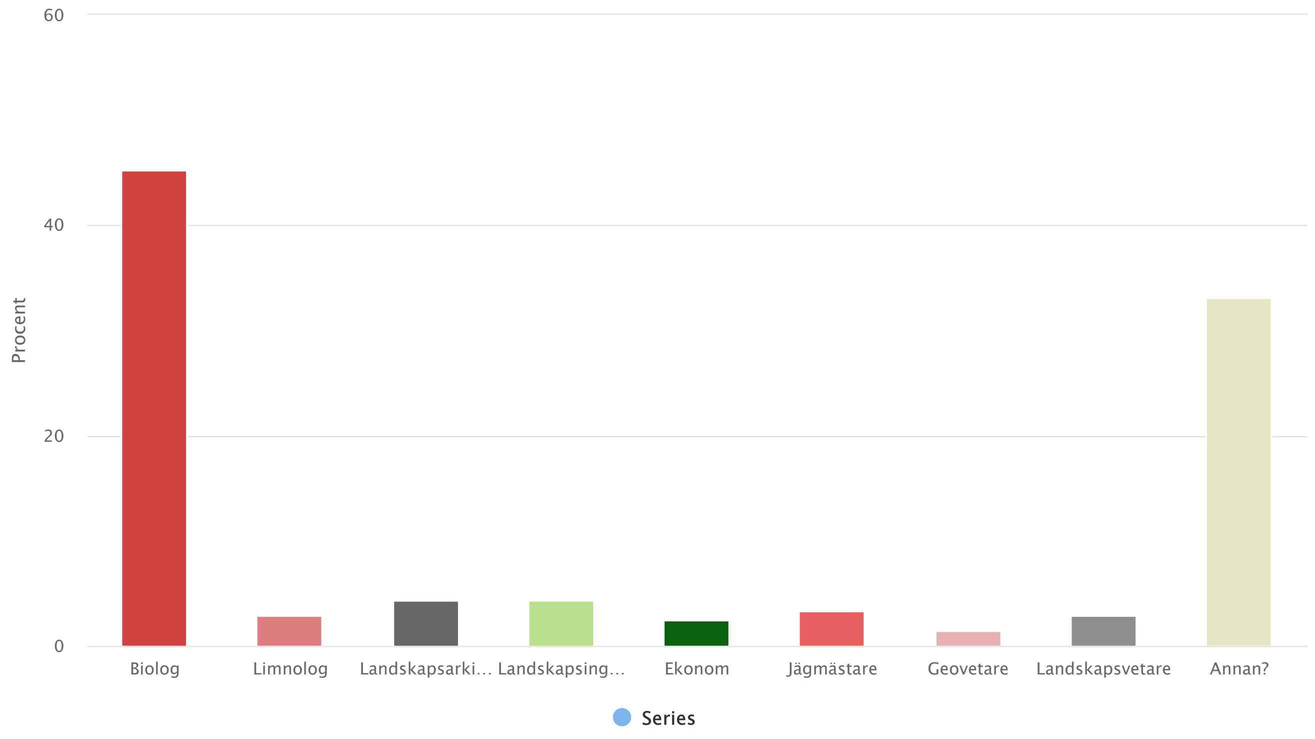

1 Biolog	94 (45%)
2 Limnolog	6 (3%)
3 Landskapsarkitekt	9 (4%)
4 Landskapsingenjör	9 (4%)
5 Ekonom	5 (2%)
6 Jägmästare	7 (3%)
7 Geovetare	3 (1%)
8 Landskapsvetare	6 (3%)
9 Annan?	69 (33%)
Svar	208

Annan?

Sida 15 - Fråga 5 | Du som förvaltare av natur och biologisk mångfald

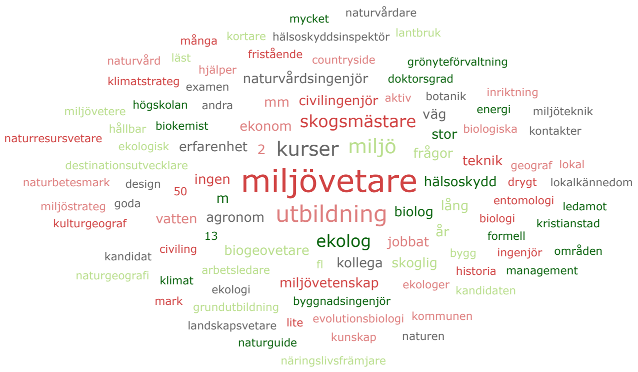

Utbildningsår?

Sida 15 - Fråga 6 | Du som förvaltare av natur och biologisk mångfald

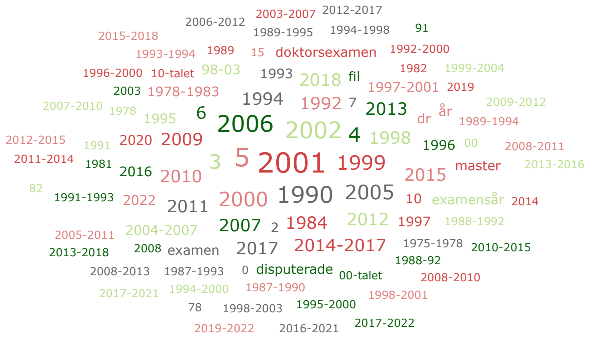

Din Ålder? Skriv en siffra

Sida 15 - Fråga 7 | Du som förvaltare av natur och biologisk mångfald

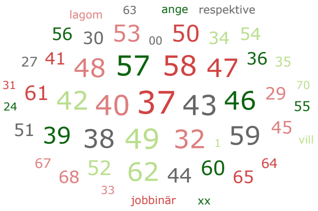

Flera kryss kan sättas.

Sida 16 - Fråga 1 | Hur tycker du att din organisation ska hantera biologisk mångfald i framtiden?

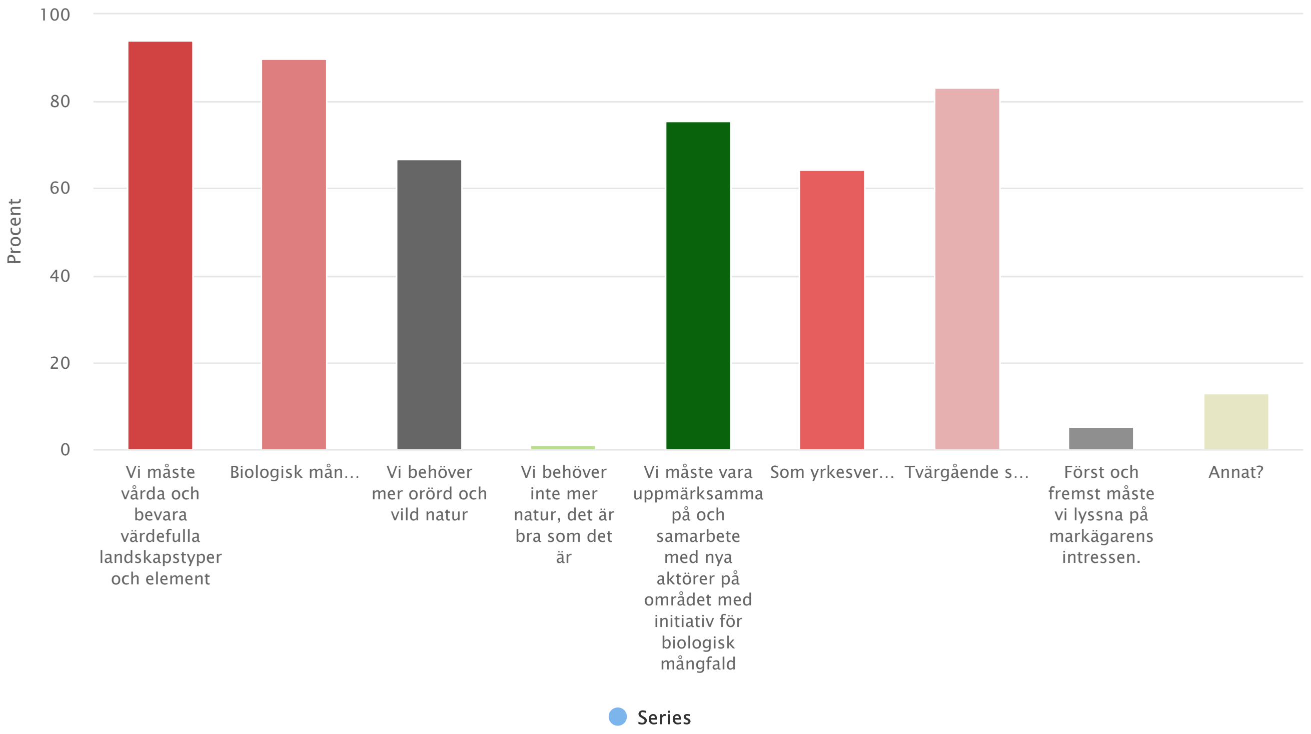

1 Vi måste vårda och bevara värdefulla landskapstyper och element	196 (94%)
2 Biologisk mångfald bör ingå som en del av multifunktionella ekosystem	187 (90%)
3 Vi behöver mer orörd och vild natur	139 (67%)
4 Vi behöver inte mer natur, det är bra som det är	2 (1%)
5 Vi måste vara uppmärksamma på och samarbete med nya aktörer på området med initiativ för biologisk mångfald	157 (75%)
6 Som yrkesverksamma är det vi som måste anstränga oss för att bromsa nedgången i den biologiska mångfalden	134 (64%)
7 Tvärgående samarbete behövs för att anpassa arbetet med biologisk mångfald till andra ekosystemtjänster	173 (83%)
8 Först och fremst måste vi lyssna på markägarens intressen.	11 (5%)
9 Annat?	27 (13%)
Svar	208

Annat?

Sida 16 - Fråga 2 | Hur tycker du att din organisation ska hantera biologisk mångfald i framtiden?

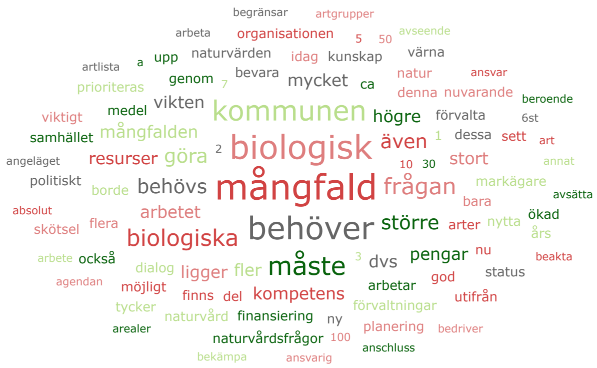

Vad känner du blir framtidens nyckelbegreppen inom biologisk mångfald förvaltning? Skriv upp till 5 ord:

Sida 17 - Fråga 1 | Din syn på förvaltning för biologisk mångfald

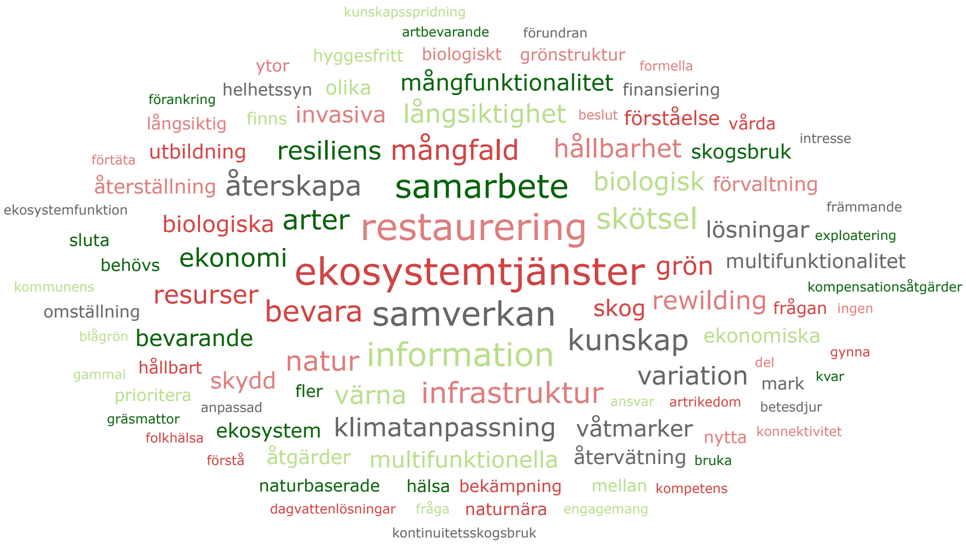

Rangordna från håller mest med (1) till håller minst med (3).

Sida 18 - Fråga 1 | Värderingar

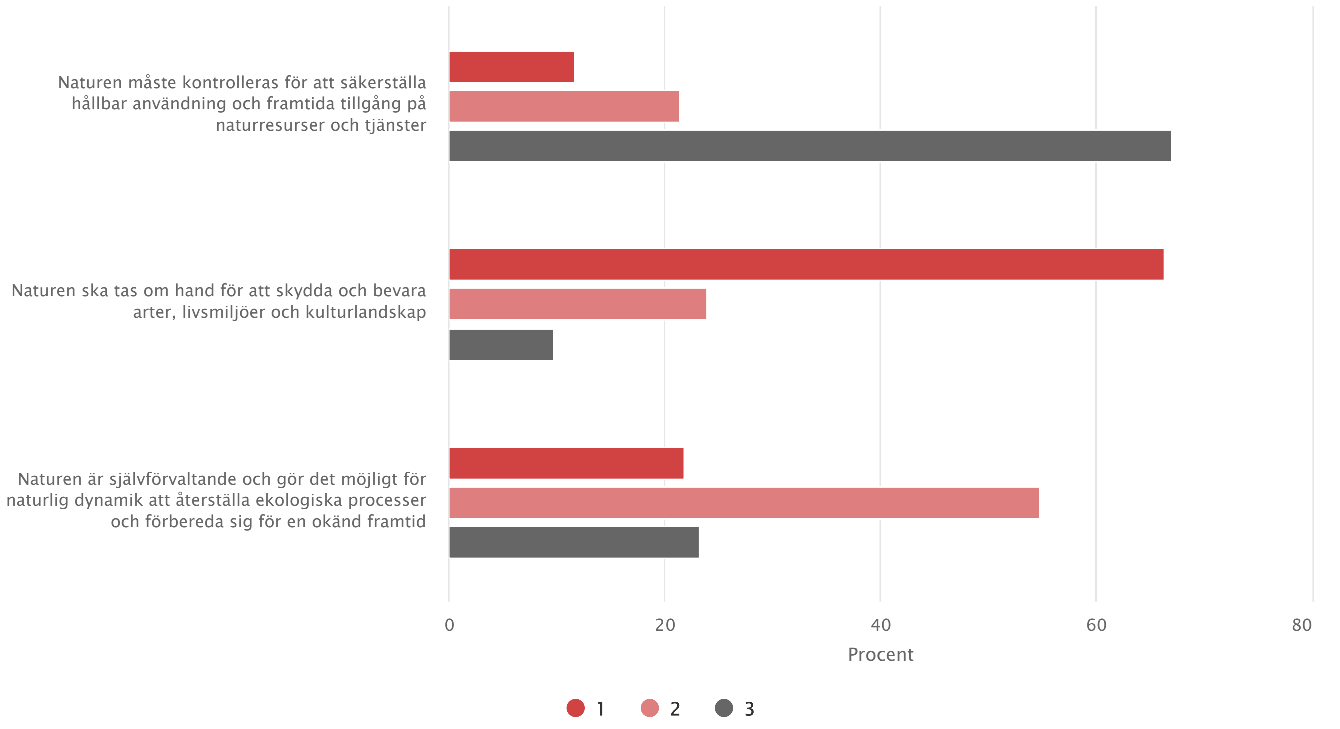

	1	2	3
Naturen måste kontrolleras för att säkerställa hållbar användning och framtida tillgång på naturresurser och tjänster	24 (12%)	44 (21%)	**138 (67%)**
Naturen ska tas om hand för att skydda och bevara arter, livsmiljöer och kulturlandskap	**136 (66%)**	49 (24%)	20 (10%)
Naturen är självförvaltande och gör det möjligt för naturlig dynamik att återställa ekologiska processer och förbereda sig för en okänd framtid	45 (22%)	**113 (55%)**	48 (23%)

	Svar
Naturen måste kontrolleras för att säkerställa hållbar användning och framtida tillgång på naturresurser och tjänster	**206**
Naturen ska tas om hand för att skydda och bevara arter, livsmiljöer och kulturlandskap	**205**
Naturen är självförvaltande och gör det möjligt för naturlig dynamik att återställa ekologiska processer och förbereda sig för en okänd framtid	**206**

Rangordna från håller mest med (1) till håller minst med (3).

Sida 19 - Fråga 1 | Värderingar

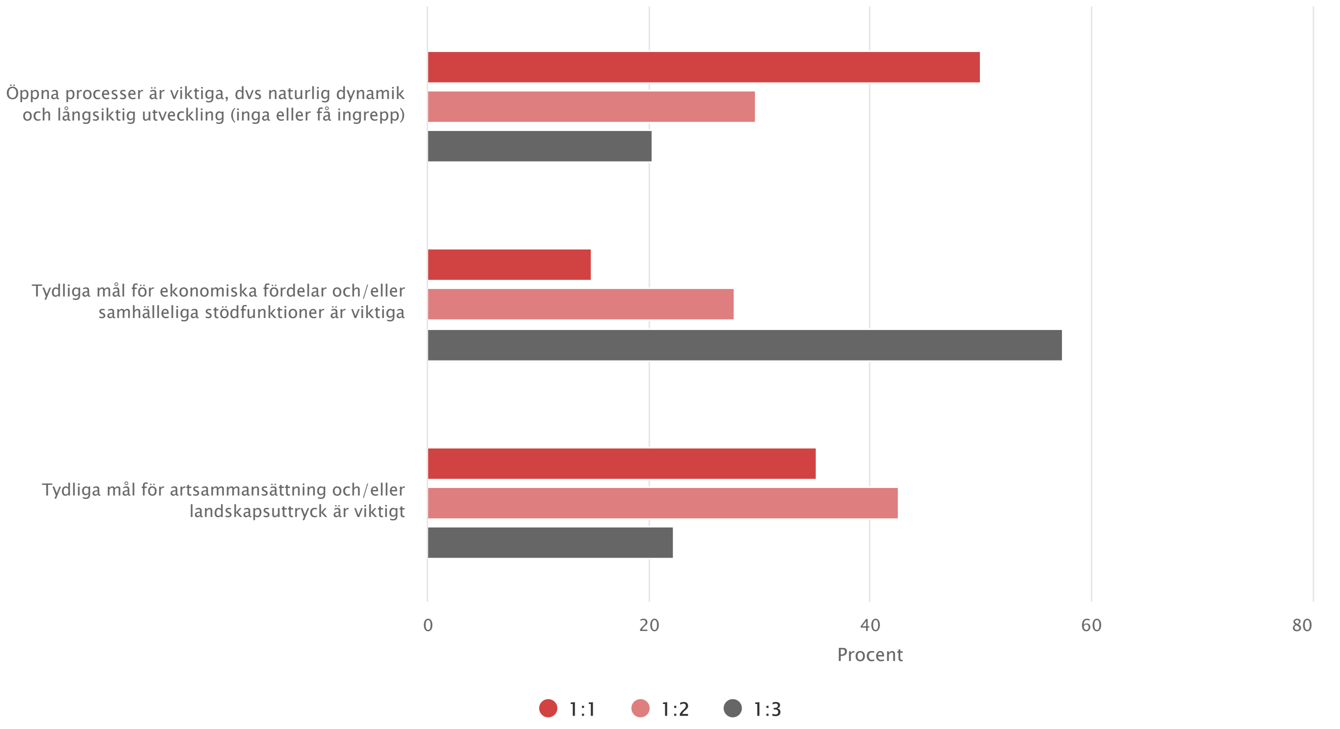

Öppna processer är viktiga, dvs naturlig dynamik och långsiktig utveckling (inga eller få ingrepp)	**101 (50%)**	60 (30%)	41 (20%)
Tydliga mål för ekonomiska fördelar och/eller samhälleliga stödfunktioner är viktiga	30 (15%)	56 (28%)	**116 (57%)**
Tydliga mål för artsammansättning och/eller landskapsuttryck är viktigt	71 (35%)	**86 (43%)**	45 (22%)

	Svar
Öppna processer är viktiga, dvs naturlig dynamik och långsiktig utveckling (inga eller få ingrepp)	**202**
Tydliga mål för ekonomiska fördelar och/eller samhälleliga stödfunktioner är viktiga	**202**
Tydliga mål för artsammansättning och/eller landskapsuttryck är viktigt	**202**

Rangordna från håller mest med (1) till håller minst med (3).

Sida 20 - Fråga 1 | Värderingar

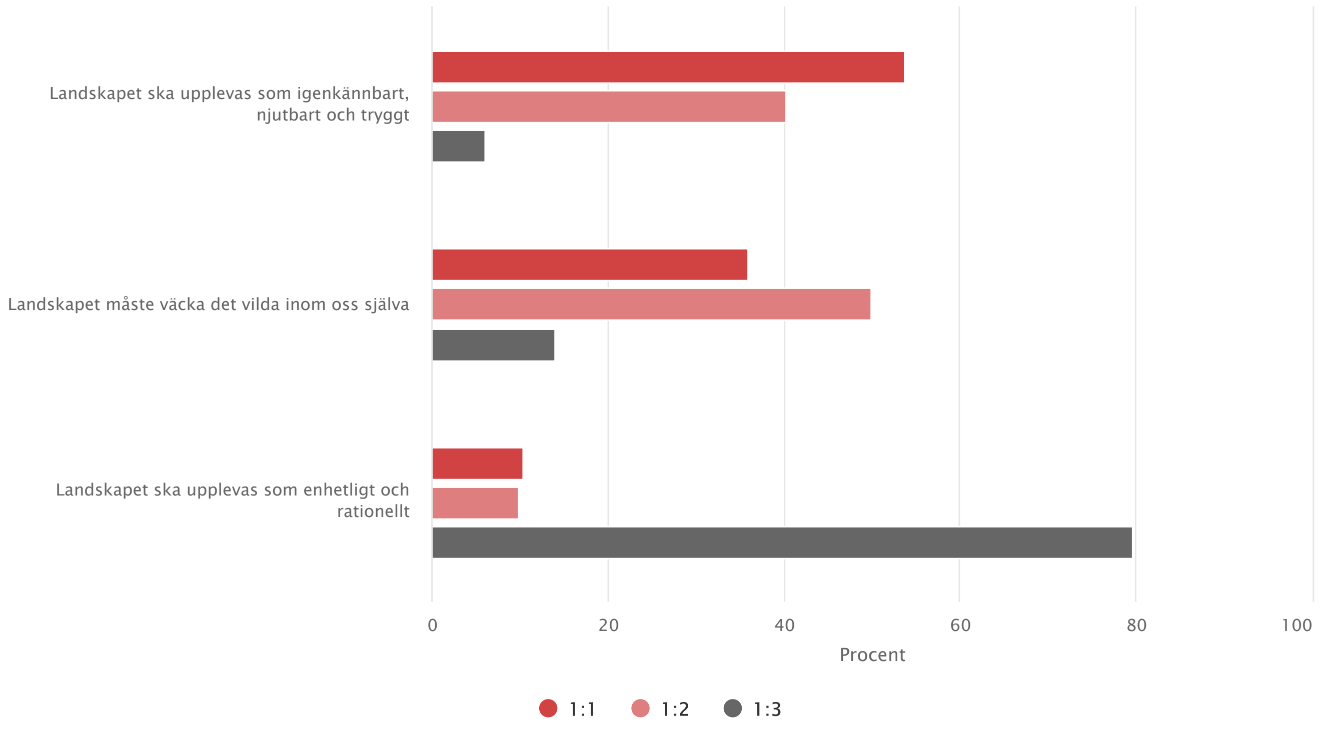

Landskapet ska upplevas som igenkännbart, njutbart och tryggt	**108 (54%)**	81 (40%)	12 (6%)
Landskapet måste väcka det vilda inom oss själva	72 (36%)	**100 (50%)**	28 (14%)
Landskapet ska upplevas som enhetligt och rationellt	21 (10%)	20 (10%)	**161 (80%)**

	Svar
Landskapet ska upplevas som igenkännbart, njutbart och tryggt	**201**
Landskapet måste väcka det vilda inom oss själva	**200**
Landskapet ska upplevas som enhetligt och rationellt	**202**

Har du ytterligare kommentarer?

Sida 21 - Fråga 1 | Tack så mycket för din insats

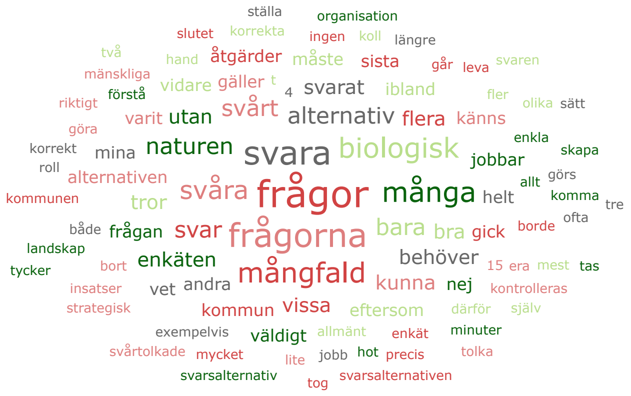

Kan vi kontakta dig med eventuella följdfrågor?

Sida 21 - Fråga 2 | Tack så mycket för din insats

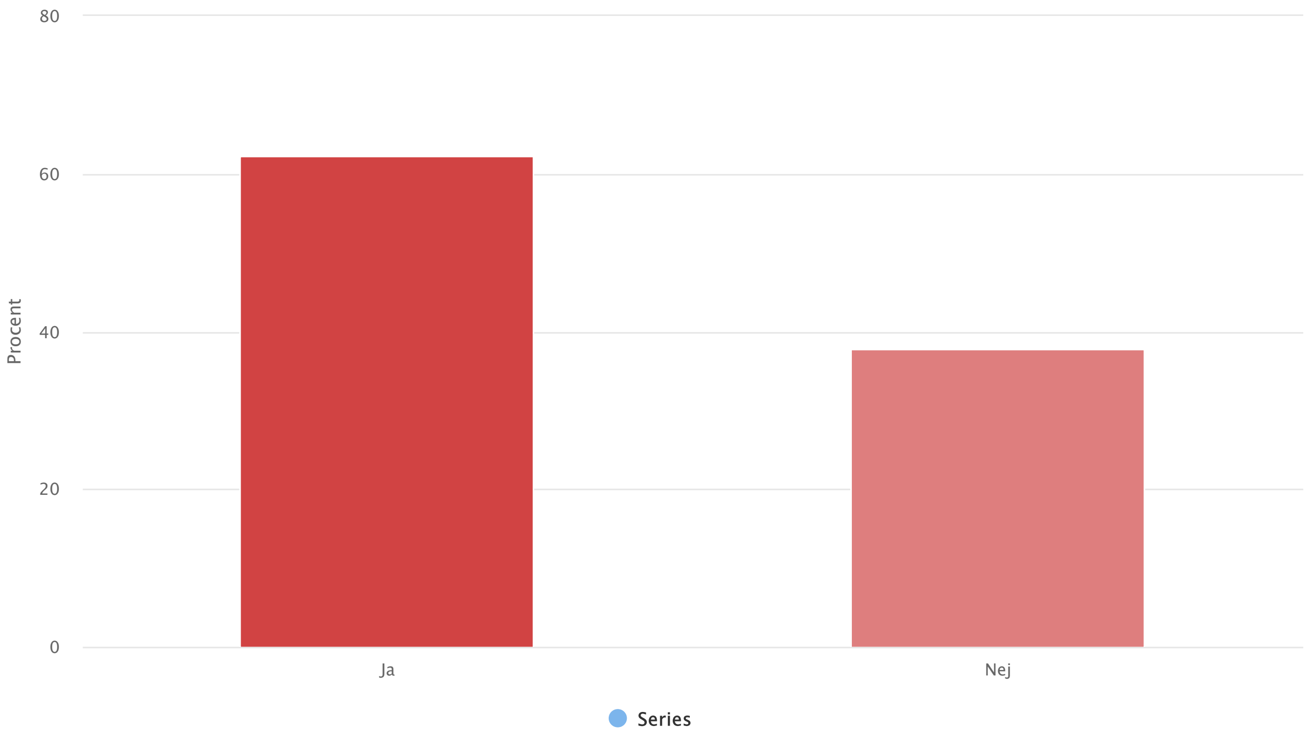

1 Ja	120 (62%)
2 Nej	73 (38%)
Svar	193
